# Boron’s Double Edge—Antibiotics, Toxins, and the Fine Line Between Them

**DOI:** 10.3390/molecules31061021

**Published:** 2026-03-18

**Authors:** Valery M. Dembitsky, Alexander O. Terent’ev, Sergey V. Baranin, Ion Romulus Scorei

**Affiliations:** 1 Bio-Pharm Laboratories, 23615 El Toro Rd X, Lake Forest, CA 92630, USA; 2 N.D. Zelinsky Institute of Organic Chemistry, Russian Academy of Sciences, 47 Leninsky Prospect, Moscow 119334, Russia; terentev@ioc.ac.ru (A.O.T.); svbar@ioc.ac.ru (S.V.B.); 3 Department of Biochemistry, BioBoron Research Institute, S.C. Natural Research S.R.L., 31B Dunării Street, 207465 Podari, Romania; romulus.scorei@naturalresearch.ro

**Keywords:** boron, borates, boronolipids, antibiotics, toxins, macrodiolides

## Abstract

Boron is a chemically distinctive bioelement whose electron-deficient structure enables reversible coordination with oxygen-rich functional groups such as diols and hydroxyls. This property allows boron to modulate molecular stability, conformation, and biological reactivity, giving rise to both beneficial pharmacological effects and toxicological outcomes. This review examines the dual biological role of boron through the framework of bioactive boron-containing natural products and natural compounds capable of forming reversible boron complexes. Particular attention is given to naturally occurring boron-containing antibiotics, including the polyketide macrodiolides boromycin, aplasmomycin, tartrolons, and hyaboron, where boron plays a direct structural and functional role in antimicrobial activity. These compounds demonstrate how boron coordination can influence ion transport, membrane interactions, and molecular assembly, contributing to potent antibacterial properties. Beyond intrinsically boron-containing metabolites, many natural antibiotics and toxins possess oxygen-rich architectures capable of forming transient borate complexes through vicinal 1,2-diol motifs. Examples include polyene macrolide antibiotics such as amphotericin B, fungichromin, and nystatin, as well as tetracyclines, rifamycins, and macrolides such as sorangicin A, where boron coordination may affect solubility, aggregation, ionophoric behavior, and biological selectivity. Similar chemistry is observed in marine neurotoxins and polyether toxins—including tetrodotoxin, saxitoxin derivatives, azaspiracids, pectenotoxins, ciguatoxins, and gambierones—whose hydroxyl-rich frameworks enable reversible interactions with boron species present in seawater. Such complexation may enhance aqueous stability and contribute to trophic transfer and bioaccumulation within marine ecosystems. By framing boron as a molecular “double edge,” this review integrates chemical, biological, and environmental perspectives to highlight how boron coordination can simultaneously enhance antimicrobial activity while influencing toxicity and ecological persistence. Recognizing the role of boron in shaping the activity of natural products provides new insight into antibiotic function, toxin behavior, and the broader impact of boron chemistry in biological systems.

## 1. Introduction

Boron is a chemically unique element whose electron-deficient structure enables the formation of reversible covalent interactions with a wide range of biological nucleophiles. These properties have made boron-containing compounds increasingly important in medicinal chemistry, where they have been successfully incorporated into therapeutics with antimicrobial, antifungal, and anti-inflammatory activities [1,2,3]. At the same time, the same reactivity that underlies their therapeutic potential can also produce significant biological toxicity, particularly when boron compounds interact with essential cellular enzymes or metabolic pathways. As a result, boron-containing molecules often occupy a narrow boundary between beneficial pharmacological agents and harmful toxins. Understanding this dual role is therefore critical for the rational design of boron-based drugs and for evaluating their biological risks [1,2,3,4,5].

Elemental boron was first isolated in 1808 by Joseph Louis Gay-Lussac, Louis Jacques Thénard, and Humphry Davy [6,7], marking the beginning of more than two centuries of research into the distinctive chemistry of this element and its growing importance in biological and pharmaceutical systems.

There is growing evidence that boron may have played a crucial role in early chemical evolution. Boron-containing lipids (boronolipids) have been proposed as contributors to the formation of primitive membranes [8,9], while boronosugars may have participated in early carbohydrate chemistry [10,11]. In addition, boron has been implicated in prebiological enantiomeric enrichment processes, potentially influencing the emergence of molecular chirality [12,13]. It is widely believed that early biological life originated in marine sedimentary environments rich in dissolved minerals, including boron [14,15,16,17,18]. Notably, the average concentration of boron in seawater is approximately 440–460 μM, which is 4.4–4.6 times higher than the concentration of dissolved iron in the ocean [19,20,21,22].

From a chemical perspective, boron typically exists in a trigonal planar BX_3_ configuration due to its 2s^2^2p^1^ electron configuration. As an electron-deficient element, boron readily accepts electron pairs through its vacant pz orbital, forming tetrahedral adducts with electron donors [23,24]. In aqueous environments, boric acid [B(OH)_3_] represents the dominant soluble form of boron in both marine and freshwater systems. Boric acid is a weak Lewis acid (pK_a_ ≈ 9.15) and exists predominantly in its undissociated form at pH values below 7. At pH values above 9, the tetrahedral metaborate anion B(OH)_4_^−^ becomes the dominant species [25,26,27,28].

At elevated boron concentrations and within the pH range of 6–11, highly water-soluble polyborate ions—such as B_3_O_3_(OH)_4_^−^, B_4_O_5_(OH)_4_^2−^, and B_5_O_6_(OH)_4_^−^—may form. However, such concentrations are rarely encountered in natural environments, rendering these species of limited biological relevance [29,30].

A defining chemical feature of boron is its strong affinity for organic molecules containing *cis*-1,2-diol motifs, leading to the formation of stable borate esters. This property underlies the biological activity of numerous boron-containing natural products and is of significant interest in bioorganic chemistry and medicinal applications [31,32,33,34].

In this review, we focus on boron-containing antibiotics and toxins produced by bacteria and other microorganisms, highlighting the dual biological roles of boron as both a therapeutic agent and a source of toxicity.

## 2. Boron-Containing Antibiotics

Antibiotics constitute a broad class of bioactive compounds produced by microorganisms or obtained through chemical modification of naturally occurring substances. These agents selectively inhibit or kill pathogenic bacteria, fungi, fungal endophytes, and, in some cases, malignant cells. As a result, antibiotics play a central role in the treatment of infectious diseases and certain cancers [35,36,37]. Many clinically important antibiotics originate from natural product scaffolds synthesized by bacteria and fungi, particularly members of the genera *Streptomyces*, *Bacillus*, and *Penicillium*. The structural diversity of these natural metabolites provides a rich source of chemically complex molecules with highly specialized biological functions. Natural product antibiotics frequently contain multiple functional groups capable of interacting with biological macromolecules, enabling selective inhibition of essential cellular processes.

The history of antibiotics began in the early 20th century with the introduction of salvarsan (arsphenamine) in 1910, developed by Paul Ehrlich for the treatment of syphilis [38,39]. Although salvarsan is an arsenic-containing compound rather than a classical antibiotic, its success marked the beginning of modern antimicrobial chemotherapy [39]. The next major milestone was the discovery of penicillin in 1929 by Alexander Fleming. Its clinical development during World War II by Ernst B. Chain and Howard W. Florey revolutionized medicine and earned the three scientists the Nobel Prize in Physiology or Medicine in 1945 [40,41,42,43]. The discovery of penicillin also demonstrated the extraordinary therapeutic potential of microbial secondary metabolites. This realization triggered extensive global efforts to isolate new antibiotics from soil microorganisms and marine environments.

The term antibiotic was introduced in 1942 by Selman A. Waksman to describe substances produced by microorganisms that inhibit the growth of other microorganisms. Over time, the definition expanded to include both naturally derived and fully synthetic antibacterial agents [44,45,46]. Within little more than a century, antibiotics have transformed clinical practice, dramatically reducing mortality from infectious diseases and increasing average human life expectancy by approximately 25 years [47,48]. Despite these advances, the emergence of antimicrobial resistance has intensified the search for new classes of bioactive compounds with novel mechanisms of action. Natural products and their derivatives continue to represent one of the most productive sources of structurally unique antimicrobial agents.

Against this historical background, boron-containing antibiotics represent a distinctive and relatively rare class of antimicrobial agents. Their biological activity is closely linked to the unique chemical properties of boron, particularly its ability to form reversible covalent bonds with biological nucleophiles [25,31,49,50,51]. This characteristic enables boron-based antibiotics to interact with essential enzymatic targets in ways that differ fundamentally from classical carbon-based drugs. In many cases, boron atoms act as electrophilic centers capable of forming transient tetrahedral complexes with hydroxyl or amino groups within enzyme active sites. Such interactions can mimic high-energy transition states in enzymatic reactions, resulting in highly potent and selective inhibition. Consequently, boron chemistry occupies a unique position at the interface between antimicrobial efficacy and potential toxicity, illustrating the dual biological role of boron explored throughout this review [49,50,51].

## 3. Boron-Containing Polyketide Antibiotics

Polyketides represent a large and structurally diverse class of natural products that include numerous clinically important antibiotics. These compounds are biosynthesized by microorganisms through the action of polyketide synthases (PKSs), large multienzyme complexes that assemble carbon skeletons from simple acyl-CoA precursors, primarily acetyl-CoA and malonyl-CoA units [52,53,54,55]. The modular and iterative nature of PKSs enables the generation of highly diverse molecular architectures, including macrolides, polyenes, and polycyclic frameworks, which confer a wide range of biological activities such as antibacterial, antifungal, immunosuppressive, and anticancer effects [55,56,57]. Many polyketide metabolites also contain extensive oxygenation patterns and polyhydroxylated domains that contribute to their chemical reactivity and biological specificity. These oxygen-rich structural motifs frequently facilitate interactions with metals or metalloid species, including boron, which can further influence molecular stability and biological function.

Boron-containing polyketides are exceptionally rare among natural products. In these compounds, boron typically occurs as a tetrahedral borate coordinated to four oxygen atoms, forming stable boron–oxygen complexes. Such borate structures are known in a variety of inorganic and organic boron compounds and have been identified in plants, cyanobacteria, and microorganisms, where they may play structural or regulatory roles [25,31,49,50,51]. The presence of boron in natural product scaffolds introduces an additional level of structural complexity and may significantly alter molecular conformation, ion-binding properties, and biological activity. Because boron can reversibly coordinate with polyhydroxylated ligands, boron-containing natural products often exhibit unique chemical stability and functional behavior compared with purely organic metabolites.

De facto, boron-containing polyketide antibiotics derived from complex fatty acid frameworks may be considered boronolipids, as they possess lipid-like polyketide architectures and form stable boron complexes via polyhydroxylated motifs [25,49,50]. These compounds typically contain macrocyclic frameworks enriched in hydroxyl groups that facilitate strong borate coordination. Such structural features allow boron to act as a central organizing element within the molecule, stabilizing the three-dimensional architecture of the macrolide framework. This boron-mediated organization can influence membrane interactions and ion transport properties that are central to their antimicrobial mechanisms.

**Figure 1:**
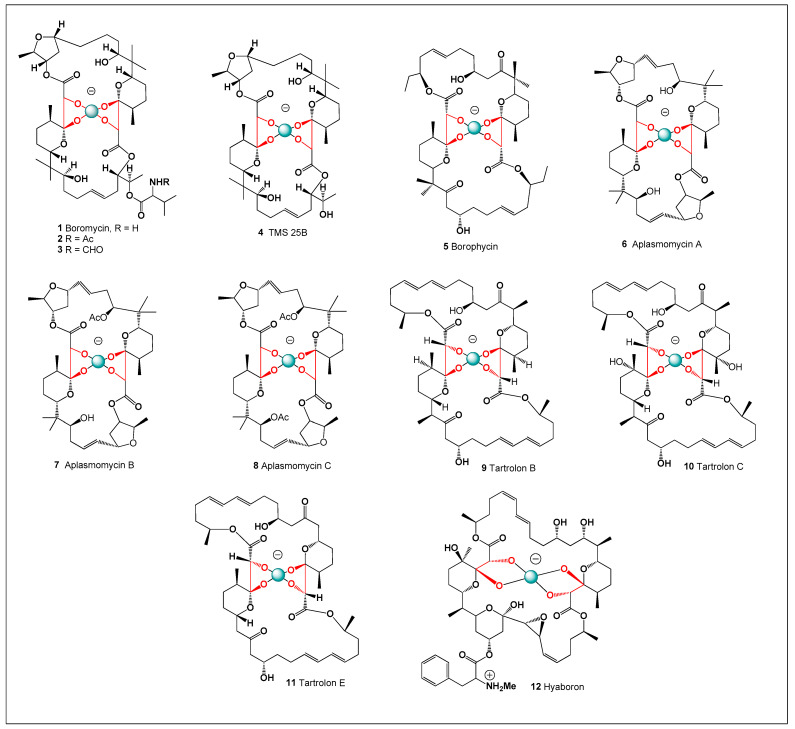
Representative structures of polyketide boron-containing antibiotics produced by marine and terrestrial microorganisms. The backbone of all the presented antibiotics is de facto a complex of two fatty acids linked by ester bonds. Highlighted in red are 1,2-diol groups, which enable the formation of boron complexes in living organisms.

**Figure 2:**
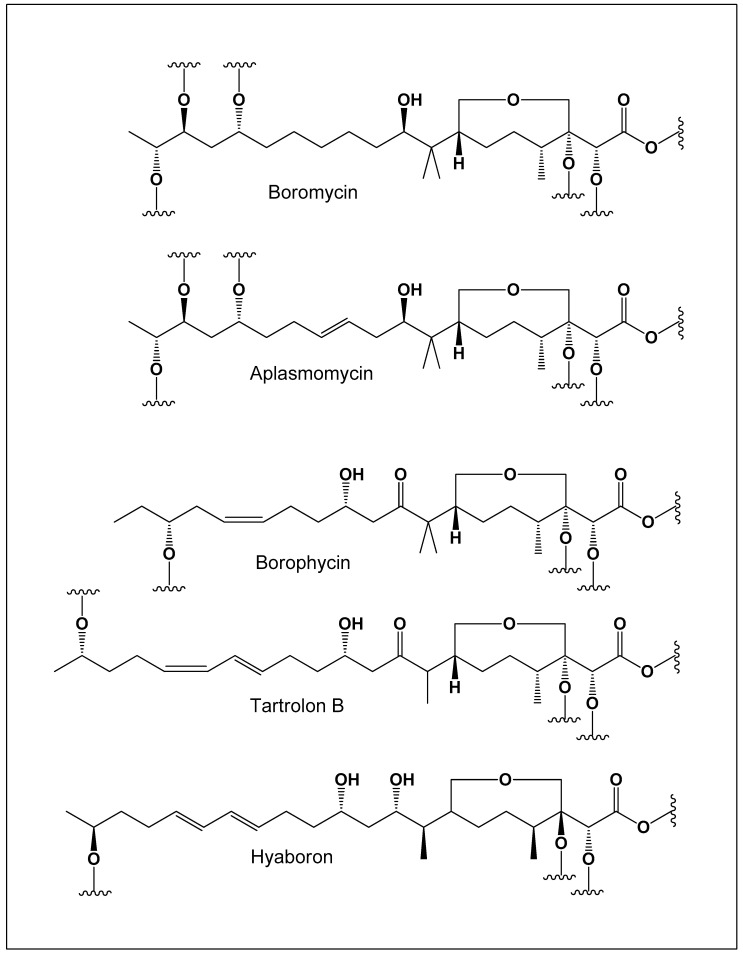
Comparative carbon skeletons of polyketide chains present in boron-containing complexes isolated from cyanobacteria, actinomycetes (Streptomycetota), and the myxobacterium *Hyalangium* spp., including borophycin, boromycin, aplasmomycin, tartrolon B, and hyaboron. The biosynthesis of boron-containing complexes appears to depend on environmental and physiological factors, including the elevated boron content of seawater, pH, temperature, and the metabolic state of the producing microorganisms. These conditions suggest that additional marine Gram-positive bacteria may be capable of producing related compounds, and that new classes of bioactive molecules, such as asymmetric boron-containing macrodiolides, are likely to be discovered in the future.

The first and most prominent example of a boron-containing polyketide antibiotic is boromycin (**1**; structures shown in Figure 1 and Figure 2), which was discovered in the 1960s from *Streptomyces antibioticus* ETH 28,829 isolated from soil samples collected in Côte d’Ivoire (Ivory Coast) [58]. Boromycin is a complex of boric acid with a tetradentate polyhydroxy macrolide ligand. Upon hydrolysis, it yields D-valine, boric acid, and a polyhydroxy macrolide-type compound, confirming its unique borate ester architecture [59,60,61,62]. Structurally, the boron atom is coordinated by oxygen atoms within the polyhydroxylated macrolide framework, forming a stable cyclic borate ester that contributes to the molecule’s conformational rigidity. This boron-centered coordination environment is essential for maintaining the biological activity of the antibiotic.

Boromycin exhibits potent antibacterial activity against Gram-positive bacteria and has also demonstrated strong antiviral activity, including inhibition of HIV replication [63,64,65]. More recently, boromycin has been shown to effectively suppress the intracellular proliferation of the protozoan parasites *Toxoplasma gondii* and *Cryptosporidium parvum*, with half-maximal effective concentrations (EC_50_) of 2.27 nM and 4.99 nM, respectively, highlighting its exceptional antiparasitic potency [66]. Mechanistic studies indicate that boromycin functions as a potassium ionophore, disrupting ion homeostasis across microbial membranes. This ion transport activity ultimately leads to membrane depolarization and cell death in susceptible organisms. The presence of the borate center is believed to play a crucial role in maintaining the structural configuration required for this ionophoric activity.

Several minor derivatives of boromycin have been identified. N-Acetylboromycin (2) and N-formylboromycin (3) were isolated from ethyl acetate extracts of *Streptomyces* sp. MA 4423 and structurally characterized by Lee and co-workers [67]. Another derivative, desvalinoboromycin (also known as TMC-25B; 4), was isolated from soil-derived *Streptomyces* species and shown to retain anti-HIV activity [59,68,69]. These derivatives demonstrate that modest structural modifications of the boromycin scaffold can preserve or alter biological activity. Continued investigation of such analogs provides insight into the structure–activity relationships governing boron-containing antibiotics. Collectively, these findings highlight the remarkable pharmacological potential of boron-based polyketides and underscore the importance of boron coordination chemistry in natural product bioactivity.

At the cellular level, boromycin has been shown to influence Ca^2+^ homeostasis in both excitable and non-excitable cells [70]. This effect is believed to arise from indirect modulation of Ca^2+^ and Na^+^ transport systems through alterations in transmembrane ionic gradients of monovalent cations. Such perturbations in ionic balance may affect multiple signaling pathways that depend on tightly regulated intracellular calcium concentrations. Because Ca^2+^ plays a central role in processes such as membrane excitability, signal transduction, and apoptosis, boromycin-induced disturbances in calcium regulation may have broad physiological consequences. Interestingly, differences in boromycin-induced Ca^2+^ transport responses may serve as a functional criterion for distinguishing excitable from non-excitable cell types.

In contrast to classical DNA-damaging agents such as bleomycin—which arrests Jurkat cells with defective G_1_ checkpoints in the G_2_ phase—and microtubule-disrupting agents such as colchicine, which block cells in the M phase [71], boromycin does not significantly alter the cell cycle profile of Jurkat cells at concentrations up to 340 nM. However, boromycin markedly potentiates the antitumor activity of bleomycin in SCID mice inoculated with Jurkat cells. This observation suggests that boromycin may sensitize tumor cells to chemotherapeutic stress without directly inducing cell-cycle arrest. The mechanism likely involves disruption of ion homeostasis that compromises cellular stress-response pathways. These findings suggest that boromycin selectively interferes with cell-cycle regulation at the G_2_ checkpoint in cancer cells, thereby enhancing the cytotoxic effects of DNA-damaging agents and highlighting its potential role as an adjuvant in anticancer therapy [71].

Boromycin was shown to strongly inhibit the replication of both clinically isolated and laboratory-adapted HIV-1 strains in in vitro experiments [72]. The antiviral mechanism of boromycin is proposed to involve interference with the late stages of the HIV replication cycle, most likely by blocking viral maturation rather than early entry or reverse transcription events. This mode of action suggests that boromycin may disrupt membrane-associated processes required for viral assembly or budding. Because viral maturation is essential for the production of infectious particles, inhibition at this stage can effectively suppress viral propagation. These observations indicate that boromycin may represent a promising scaffold for the development of antiviral agents targeting late stages of viral replication.

In bacterial systems, boromycin at a concentration of 0.05 μg/mL inhibits the synthesis of proteins, RNA, and DNA in whole cells of *Bacillus subtilis* [73]. Its antibacterial activity is antagonized by surface-active compounds, and the antibiotic readily binds to lipoproteins. Within bacterial cells, boromycin preferentially associates with the cytoplasmic membrane. Notably, the inhibitory effect on *B. subtilis* can be reversed by high concentrations of potassium salts (e.g., 0.2 M KCl), an effect that is specific to potassium ions. Addition of boromycin induces a rapid efflux of intracellular K^+^ ions, while the K^+^/Na^+^-activated ATPase of the cytoplasmic membrane remains unaffected. These observations indicate that boromycin acts as a potassium ionophore, disrupting ionic homeostasis rather than directly inhibiting ATPase activity. The resulting collapse of potassium gradients leads to membrane depolarization and loss of essential cellular functions. Such ionophoric activity is consistent with the macrocyclic structure of boromycin and its ability to coordinate ions within its polyoxygenated framework. Furthermore, degradation of boromycin by either acidic or alkaline hydrolysis leads to loss of antibiotic activity, consistent with cleavage of the boric acid moiety from the molecule, underscoring the critical role of the boron center in biological function [74,75].

A structurally related boron-containing analog, borophycin (5), was isolated from marine strains of the cyanobacteria *Nostoc linckia* and *Nostoc spongiaeforme* var. *tenue*. Borophycin exhibits potent cytotoxic activity against human epidermoid carcinoma and colorectal adenocarcinoma cell lines and also displays antimicrobial properties [76,77,78,79,80]. Structurally, borophycin shares key features with other boron-containing macrolides, including extensive oxygenation and the ability to coordinate boron within a polyhydroxylated framework. These structural characteristics may contribute to its strong interactions with biological membranes and intracellular targets. The discovery of borophycin in marine cyanobacteria also highlights aquatic environments as an important source of unusual boron-containing natural products. Its biological activity further underscores the pharmacological potential of boron-containing macrolide structures in antitumor and antimicrobial drug discovery.

Another notable boron-containing antibiotic, aplasmomycin (6), was isolated from a marine strain of *Streptomyces griseus* obtained from shallow sea sediments in Sagami Bay. Aplasmomycin inhibits the growth of Gram-positive bacteria, including mycobacteria, in vitro, and exhibits antiplasmodial activity in vivo [81,82]. Two closely related analogs, aplasmomycin B (7) and aplasmomycin C (8), were subsequently discovered from the same microbial strain [83], and the biosynthesis of aplasmomycin has been elucidated in detail [84]. Structurally, aplasmomycin belongs to the family of boron-containing macrodiolide antibiotics, in which a central borate moiety is coordinated by multiple oxygen atoms within a polyhydroxylated macrolide framework. This boron-centered coordination plays a key role in stabilizing the three-dimensional conformation of the molecule and contributes to its ion-binding properties.

Given that ionophore antibiotics are capable of transporting cations across lipid membranes, aplasmomycin and its analogs were systematically compared with respect to antibacterial activity, alkali metal ion selectivity, and potassium transport capacity. The antibacterial activity of aplasmomycin B (7) was found to be comparable to that of aplasmomycin, whereas aplasmomycin C (8) exhibited reduced potency. Deboro-aplasmomycin showed even weaker activity than aplasmomycin C. The alkali metal ion selectivity of these compounds followed the order Rb^+^ > K^+^ > Cs^+^ ≈ Na^+^ > Li^+^, with no detectable affinity for divalent cations. Importantly, the relative cation affinities did not correlate directly with antibacterial potency [82,83,84,85]. These findings indicate that subtle structural variations around the boron coordination environment can significantly influence biological activity.

In potassium transport assays using a modified Pressman system, both aplasmomycin and aplasmomycin B efficiently mediated net K^+^ transport through the bulk phase, whereas aplasmomycin C and deboro-aplasmomycin were incapable of transporting potassium ions. This observation closely mirrors their respective antibacterial activities and supports the conclusion that potassium ionophore activity is a key determinant of the biological effects of aplasmomycin and its analogs [82]. The ability to selectively transport potassium ions across biological membranes disrupts intracellular ionic balance and ultimately compromises cell viability. Such ionophoric mechanisms are common among macrocyclic antibiotics with extensive oxygenation patterns capable of coordinating metal ions.

Aplasmomycin (**6**) was obtained from a strain of *Streptomyces griseus* isolated from shallow marine sediments in Sagami Bay, Japan [86,87,88,89]. The producing strain originated from a nutrient-poor environment characterized by relatively high salinity, prompting detailed studies on the influence of nutrient availability and sodium chloride concentration on aplasmomycin biosynthesis. Cultivation experiments were conducted using a basal medium containing yeast extract (0.4%), malt extract (1.0%), and glucose (0.4%) at pH 7.4. The antibiotic was preferentially produced under conditions of diluted nutrients combined with elevated salt concentrations [82,87,88,89]. These observations suggest that environmental stress conditions may stimulate the biosynthesis of secondary metabolites such as aplasmomycin in marine microorganisms.

Aplasmomycin exhibits potent antibacterial activity against Gram-positive bacteria, including mycobacteria. In addition, it shows strong in vivo antiplasmodial activity. Male mice infected intraperitoneally with *Plasmodium berghei* were treated orally with aplasmomycin formulated in peanut oil, resulting in a marked reduction in parasitized erythrocytes and complete survival of treated animals. This pronounced antiplasmodial efficacy served as the basis for naming the compound. Acute toxicity studies revealed an LD_50_ value of approximately 125 mg/kg following intraperitoneal administration in mice [81,82,83,84]. These pharmacological properties highlight the potential of aplasmomycin as a lead compound for the development of new antimicrobial and antiparasitic therapies.

The structure of the silver salt of aplasmomycin was elucidated by X-ray crystallographic analysis [90,91,92], and the molecular structure was further confirmed by ^1^H and ^13^C NMR spectroscopy [93,94]. Notably, the conformation of aplasmomycin in CDCl_3_ solution was found to be identical to that observed in the solid state. Removal of the boron atom from the molecule resulted in only minor conformational changes, indicating that the boron moiety plays a critical functional rather than structural role. This observation suggests that boron contributes primarily to the molecule’s ion-binding and biological properties rather than serving as a rigid structural scaffold. Such findings further emphasize the unique chemical role of boron in modulating the activity of natural product antibiotics.

Under the same cultivation conditions, *Streptomyces griseus* also produced two minor analogs, aplasmomycins B and C [83]. These compounds share the same macrodiolide framework as aplasmomycin but differ slightly in substitution patterns that influence their ion-binding properties and biological potency. More recently, an actinomycete capable of producing aplasmomycin C was isolated from sandy marine sediments collected off the coast of California [95], highlighting the broader environmental distribution of boron-containing ionophore antibiotics. The occurrence of these compounds in geographically distant marine environments suggests that boron-utilizing biosynthetic pathways may be more widespread among actinomycetes than previously recognized. Such findings support the idea that marine and sediment-associated microorganisms represent a significant reservoir of structurally unusual boron-containing natural products.

An asymmetric boron-containing macrodiolide, named hyaboron (12), was isolated from the myxobacterium *Hyalangium minutum*, a mesophilic soil-dwelling microorganism originally obtained from decaying wood. Functional studies demonstrated that hyaboron, along with other naturally occurring borated compounds, induces inflammasome-dependent maturation of interleukin-1β (IL-1β) via activation of the NLRP3 inflammasome. This effect is most likely associated with their ability to function as potassium ionophores, leading to intracellular K^+^ efflux—an established trigger for inflammasome activation. The resulting activation of inflammatory signaling pathways suggests that boron-containing ionophores may influence host immune responses in addition to exerting antimicrobial effects. In addition to its immunomodulatory activity in both human and murine cells, hyaboron (12) exhibits a broad spectrum of biological activities, including antibacterial and antiparasitic effects [96,97].

Structurally, all known polyketide boron-containing antibiotics are composed of two asymmetric or symmetric fatty acid–derived units linked through ester bonds, forming macrocyclic or macrodiolide frameworks (see Figure 2). These polyoxygenated structures provide multiple coordination sites capable of stabilizing a central borate ion within the macrocyclic cavity. The resulting boron–oxygen coordination contributes to the conformational stability and ion-binding properties that underlie the biological activity of these compounds. Such structural arrangements are characteristic of boron-containing ionophore antibiotics and distinguish them from conventional macrolide natural products. The biological activities of these boron-containing antibiotics have been extensively investigated and are well documented in both experimental and review literature [49,50,51].

Applying the principle of mathematical induction, which allows generalization from individual observations to broader laws, we propose that additional microorganisms are likely capable of synthesizing polyketide boron-containing antibiotics [98,99,100]. This biosynthetic capability is expected to emerge particularly in environments where boron is quantitatively abundant relative to iron or other metals, such as seawater or boron-rich soils, and in microorganisms that have developed boron resistance or tolerance mechanisms. Microbial adaptation to elevated boron concentrations may favor the evolution of metabolic pathways that incorporate boron into secondary metabolites. Such adaptations could provide ecological advantages by enabling organisms to produce chemically unique antimicrobial compounds. Continued exploration of diverse microbial habitats is therefore likely to reveal additional boron-containing natural products and expand our understanding of boron-based biosynthetic chemistry.

To further support this hypothesis, an analysis of the ChemNetBase database (Taylor & Francis (Boca Raton, FL, USA)) identified numerous natural and synthetic compounds containing *cis*-1,2-diol motifs, which are well known to form stable complexes with boron (see Figure 1). These structural features are particularly common in polyhydroxylated natural products, including polyketides, polyenes, and polyether toxins, where adjacent hydroxyl groups provide ideal coordination sites for borate formation. The ability of boron to reversibly bind such diol motifs enables the formation of cyclic borate esters that can significantly alter molecular conformation and physicochemical behavior. Moreover, exploration of large-scale graphical chemical repositories such as ChemSpace, which contains over 13 billion chemical building blocks, revealed a substantial number of molecular scaffolds with the structural potential to give rise to novel boron-containing antibiotics or toxins. Many of these scaffolds possess polyoxygenated frameworks that could support boron coordination under appropriate environmental or biochemical conditions. This observation suggests that boron-mediated chemistry may be far more prevalent in natural product systems than is currently recognized. These findings highlight a vast and largely unexplored chemical space for the discovery of new boron-based bioactive compounds.

In addition to aplasmomycin, tartrolon B (9), C (10), and E (11) polyketide antibiotics were isolated from the culture broth of the gliding bacterium *Sorangium cellulosum* strain So ce678 [101,102,103,104]. These macrodiolide compounds share structural similarities with other boron-associated polyketide ionophores and contain polyoxygenated frameworks capable of coordinating boron through multiple hydroxyl groups. These compounds exhibit antibacterial activity against Gram-positive bacteria and display cytotoxic effects toward mammalian cells [49,50]. The biological activity of tartrolons is believed to be associated with their ability to transport monovalent cations across biological membranes, thereby disrupting ionic homeostasis. Borated tartrolon analogs were subsequently identified in symbiotic cellulose-degrading bacteria (*Teredinibacter turnerae*) residing in shipworm gills [104]. The presence of these compounds in marine symbiotic bacteria suggests that boron-associated polyketide metabolites may play ecological roles in microbial competition and host–microbe interactions.

*Sorangium cellulosum* is a cellulose-degrading myxobacterium originally isolated from soil in 1937 by Imshenetski and Solntseva. Remarkably, strains of *S. cellulosum* are the only known myxobacteria capable of degrading crystalline cellulose and utilizing it as a sole carbon source. Members of the genus *Sorangium* are attracting increasing scientific interest due to their complex life cycles, exceptionally large genomes—the largest among known bacteria—and their outstanding capacity to produce structurally diverse and biotechnologically valuable secondary metabolites [101].

**Figure 3:**
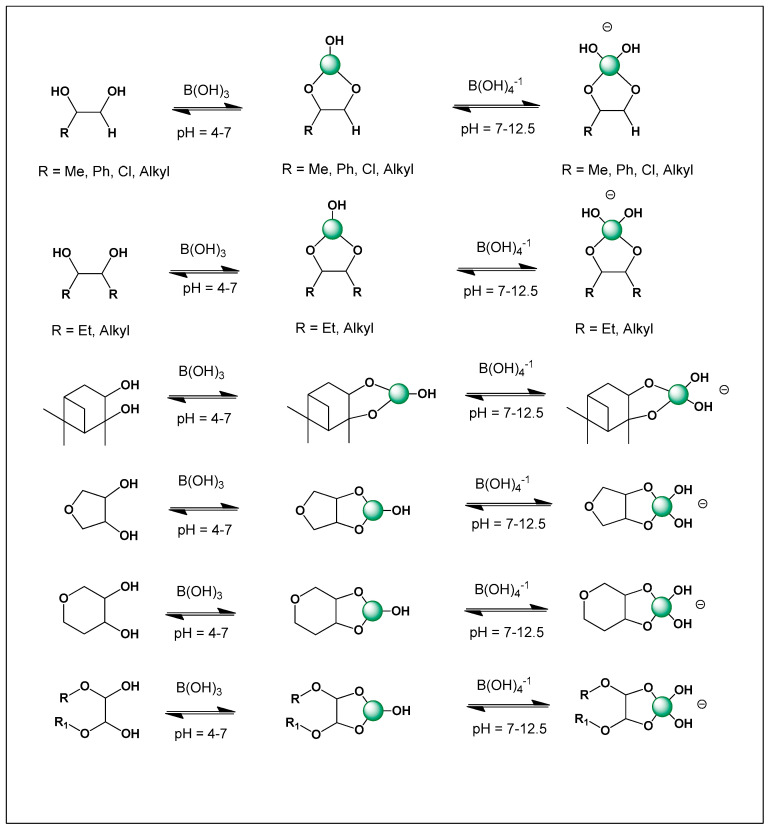
Natural antibiotics and toxins containing 1,2-*cis*-diol moieties are capable of forming trigonal planar and/or tetrahedral borate complexes. Depending on their biological effects on microorganisms or higher organisms, these boron complexes may be classified as antibiotics or toxins. Tri-(trigonal planar (BO_3_)) and tetrahedral (BO_4_) units are the fundamental building blocks of borate complexes, which link via shared oxygens to form diverse structures like chains, rings (triborates, tetraborates), and complex anions (e.g., (B_2_O_5_)^−4^, (B_4_O_12_)^−12^) found in minerals and glasses, with their ratio determining the overall structure. Boron’s ability to coordinate with three or four oxygens leads to varied chemistries, from simple borate ions to sophisticated ligands in catalysis and materials.

Figure 3 shows the structures of natural antibiotics and toxins containing cis-1,2-diol moieties, which are capable of forming trigonal planar and/or tetrahedral borate complexes, depending on the pH of the medium and the presence of boric acid or boron anion. Furthermore, depending on their biological effects on microorganisms or higher organisms, these boron complexes can be classified as antibiotics or toxins.

*Sorangium cellulosum* is a cellulose-degrading myxobacterium originally isolated from soil in 1937 by Imshenetski and Solntseva. Remarkably, strains of *S. cellulosum* are the only known myxobacteria capable of degrading crystalline cellulose and utilizing it as a sole carbon source. Members of the genus *Sorangium* are attracting increasing scientific interest due to their complex life cycles, exceptionally large genomes—the largest among known bacteria—and their outstanding capacity to produce structurally diverse and biotechnologically valuable secondary metabolites [101]. The remarkable biosynthetic capabilities of these organisms are reflected in the large number of polyketide synthase gene clusters present in their genomes. These biosynthetic pathways enable the production of chemically complex natural products with diverse pharmacological properties.

Figure 3 shows the structures of natural antibiotics and toxins containing cis-1,2-diol moieties, which are capable of forming trigonal planar and/or tetrahedral borate complexes, depending on the pH of the medium and the presence of boric acid or borate anions. The formation of such borate complexes can influence the stability, solubility, and biological activity of these molecules. In aqueous environments, reversible borate ester formation with vicinal diols may alter the conformational flexibility and membrane interactions of these compounds. Furthermore, depending on their biological effects on microorganisms or higher organisms, these boron complexes can be classified as antibiotics or toxins. This dual functional behavior illustrates the fine line between beneficial antimicrobial activity and toxic effects that characterizes many boron-associated natural products.

### 3.1. Structural Features, Formation, and Biological Activity of Boron-Containing Polyketide Antibiotics

Boron-containing polyketide antibiotics such as boromycin, borophycin, aplasmomycin, tartrolons, and hyaboron share a set of distinctive structural features that strongly suggest common principles governing their formation and biological activity. Structurally, these compounds are characterized by macrocyclic polyketide frameworks rich in oxygenated functional groups, particularly vicinal cis-1,2-diols and polyhydroxy motifs. These functionalities provide ideal coordination sites for boron, allowing the formation of neutral borate esters or tetrahedral boron complexes stabilized by intramolecular chelation.

#### 3.1.1. Possible Formation Mechanisms

The biosynthesis of the polyketide backbone in these antibiotics follows canonical type I or type II polyketide synthase (PKS) pathways, producing highly oxygenated macrolide or macrodiolide scaffolds. Boron incorporation is unlikely to be enzyme-mediated in the classical sense; instead, available evidence suggests a post-biosynthetic, spontaneous complexation mechanism. In boron-rich environments—such as marine sediments, seawater, or boron-enriched soils—polyketide intermediates containing appropriately oriented diol groups can chelate boric acid or borate ions present in the surrounding medium.

This hypothesis is supported by several observations: (i) boron is not covalently embedded into carbon frameworks but exists as a reversible coordination complex; (ii) removal of boron often leads to loss or attenuation of biological activity, indicating a functional role rather than a structural artifact; (iii) similar polyketide scaffolds lacking boron are widely distributed, suggesting that boronation depends on environmental availability rather than specialized genetics alone.

Thus, boron incorporation may represent an adaptive chemical modification, emerging at the interface between microbial metabolism and environmental chemistry.

#### 3.1.2. Structure–Activity Relationships

The biological activity of boron-containing polyketide antibiotics is closely tied to their three-dimensional architecture and ionic properties, both of which are influenced by boron coordination. Formation of borate complexes can: (i) increase molecular rigidity, stabilizing bioactive conformations; (ii) modify lipophilicity and charge distribution, enhancing membrane permeability; (iii) enable cation-binding and ionophoric behavior, particularly toward K^+^ and Na^+^ ions.

Indeed, many boron-containing polyketide antibiotics act as ionophores, disrupting cellular ion gradients and leading to rapid loss of membrane potential. This mechanism explains their strong activity against Gram-positive bacteria, parasites, and cancer cells, all of which are highly sensitive to ionic imbalance. In compounds such as boromycin, aplasmomycin, and hyaboron, biological activity correlates with potassium efflux, in flamma some activation, and apoptosis induction.

#### 3.1.3. Boron as a Functional Amplifier

Rather than serving as a passive structural appendage, boron appears to function as a biological activity amplifier. By enabling reversible complexation, boron confers a unique duality: (i) selective toxicity toward microbial or malignant cells, due to differences in membrane composition and ion homeostasis; (ii) context-dependent activity, influenced by pH, ionic strength, and boron availability.

This duality aligns with the broader concept of boron as a “double-edged” element—capable of enhancing antibiotic potency under favorable conditions while also contributing to cytotoxic or immunomodulatory effects.

#### 3.1.4. Evolutionary and Ecological Implications

The recurring appearance of boron-containing polyketides in phylogenetically distant microorganisms—cyanobacteria, actinomycetes, and myxobacteria—suggests convergent chemical evolution rather than shared ancestry. In boron-rich niches, microorganisms capable of producing highly oxygenated polyketides gain access to an additional chemical dimension: boron-mediated bioactivity. This may confer a competitive advantage in microbial ecosystems by expanding the functional repertoire of secondary metabolites without requiring new enzymatic machinery.

## 4. Polyene Macrolide Antibiotics

Polyene macrolide antibiotics are potent, naturally derived antifungal agents (e.g., Amphotericin B and Nystatin) that act by binding to ergosterol in fungal cell membranes and forming transmembrane pores that lead to ion leakage and cell death, making them indispensable for the treatment of severe systemic mycoses, despite their well-known toxicity [105,106,107]. Structurally, these compounds contain a large macrolactone ring with multiple conjugated double bonds forming the characteristic “polyene” chromophore and are biosynthesized primarily by bacteria of the genus *Streptomyces*, often bearing glycosidic substituents that influence solubility and biological activity. Their mechanism of action involves high-affinity interaction with ergosterol, the fungal analog of cholesterol, resulting in disruption of membrane integrity and leakage of essential intracellular electrolytes, ultimately causing fungal cell death. Polyene macrolides are therefore strongly fungicidal and exhibit activity against a broad range of clinically significant pathogens, including *Candida*, *Aspergillus*, *Cryptococcus*, and *Histoplasma*, and in some cases show activity against protozoan parasites [107,108,109,110]. In addition to their well-established membrane-targeting activity, the highly oxygenated frameworks of many polyene macrolides contain vicinal diol and polyol motifs capable of coordinating metal ions or forming reversible complexes with boron species. Such interactions may influence aggregation behavior, ionophoric properties, and membrane selectivity, providing a potential chemical basis for variations in antifungal potency and host toxicity. These structural features also make polyene macrolides relevant to discussions of boron-mediated complexation, as transient borate interactions could modulate their physicochemical behavior in biological or environmental systems.

### Amphotericin B and Its Boron Complexes

**Figure 4:**
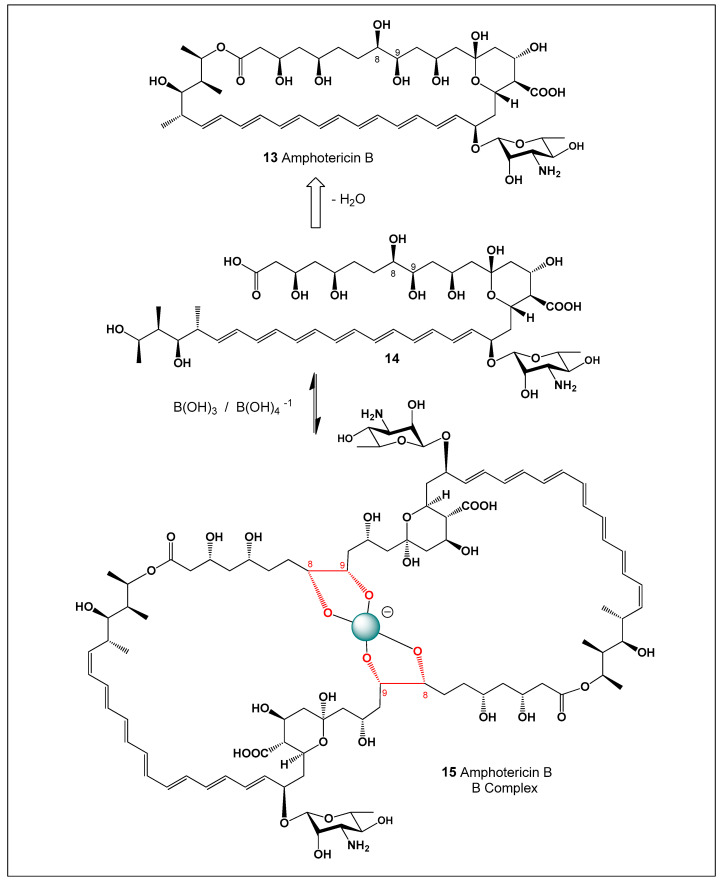
Proposed mechanistic pathways for the formation of amphotericin B and its boron complexes. A linear polyhydroxy polyketide precursor undergoes enzyme-mediated macrolactonization accompanied by stepwise dehydration reactions, yielding the cyclic polyene macrolide amphotericin B. In parallel, the same polyhydroxylated scaffold contains vicinal cis-diol motifs capable of reversibly chelating boric acid or borate anions, forming tetrahedral boron–diolate complexes. This boron coordination can occur both at the precursor stage and on the mature amphotericin B molecule, leading to borate-bridged assemblies or copolymeric chains. The equilibrium between free and boron-complexed forms is pH-dependent and influences molecular aggregation, conformation, and solubility, providing a mechanistic link between polyene macrolide structure, boron chemistry, and biological activity modulation.

Amphotericin B (AmB, **13**) was first isolated in 1955 from a strain of *Streptomyces nodosus* collected along the banks of the Orinoco River in Venezuela [111,112,113,114]. Owing to its exceptionally potent antifungal activity, AmB was rapidly introduced into clinical practice and has since remained a cornerstone therapy for the treatment of life-threatening systemic mycoses. AmB and all polyene macrolide antibiotics are formed from a polyketide precursor (**14**) by ester bond formation (see Figure 4).

The molecular structure of amphotericin B was unambiguously elucidated in 1970 by Schaffner and co-workers, revealing the structural basis for its pronounced amphiphilic character [115,116,117]. The molecule consists of a rigid macrolide framework bearing two clearly differentiated domains: a hydrophobic polyene region and a hydrophilic polyol region, which are arranged longitudinally along the molecular axis. In addition, a polar amino sugar moiety, known as mycosamine, is attached to the macrolide ring and plays a critical role in sterol recognition. At physiological pH, both the amino group of the mycosamine unit and the carboxylic acid of the macrolide are ionized, with reported pK_a_ values of approximately 10.0 and 5.5, respectively [118,119].

Approximately a decade after its discovery, the first mechanistic investigations into the mode of action of amphotericin B were undertaken [120,121]. These pioneering studies demonstrated that exposure of fungal cells to AmB leads to profound alterations in membrane permeability, resulting in the leakage of mono- and divalent ions—most notably potassium ions (K^+^), but also calcium (Ca^2+^), magnesium (Mg^2+^), and phosphate (PO_4_^3−^)—as well as other low-molecular-weight cellular constituents. These ionic disturbances were accompanied by inhibition of essential metabolic processes, including glycolysis and protein synthesis [122].

The close temporal association between membrane permeabilization, metabolic inhibition, and cell death led to the proposal that amphotericin B exerts its antifungal activity primarily through the formation of transmembrane channels or pores in sterol-containing membranes [123,124]. This pore-forming mechanism, driven by selective interactions with ergosterol in fungal membranes, remains one of the most widely accepted explanations for the potent antifungal efficacy of amphotericin B and underpins both its therapeutic success and its dose-limiting toxicity. In fungal cells, the insertion of amphotericin B aggregates into the membrane disrupts ion gradients by allowing uncontrolled leakage of intracellular ions such as K^+^, Mg^2+^, and small metabolites. Amphotericin B, a prototypical polyene macrolide antibiotic, exists in aqueous solution predominantly as poorly soluble, high–molecular-weight aggregates, which strongly influence its pharmacological behavior. To address these limitations, a borate complex of amphotericin B was prepared and shown to exhibit markedly enhanced solubility and reduced aggregation relative to the parent compound. Such borate complexation is thought to involve coordination between borate ions and the multiple hydroxyl groups present in the polyol region of amphotericin B. In aqueous media, this borate complex does not exist as a single discrete species but rather as a heterogeneous mixture of molecular assemblies that differ in borate content, molecular weight, and conformational organization [123,124,125].

The solubility of the amphotericin B–borate complex (15) is strongly pH-dependent, reaching a minimum near neutral pH, yet remaining one to two orders of magnitude higher than that of native amphotericin B across the entire pH range studied. This pronounced pH dependence reflects the equilibrium between boric acid and borate anions in aqueous solution, which governs the extent of complex formation. Analysis of molecular size distribution by differential ultrafiltration revealed a progressive increase in the fraction of aggregated species as the pH shifted from acidic to alkaline conditions. The aggregates ranged broadly in size, from fewer than 25 amphotericin B units to assemblies containing more than 100 molecules [126,127,128]. Such large supramolecular assemblies are characteristic of polyene antibiotics and play a key role in determining their biological and physicochemical properties.

The borate content of the complexes increased systematically with increasing pH, while no borate incorporation was detected under acidic conditions. These observations indicate that amphotericin B and borate ions form copolymeric chains of variable length in which the two components alternate. Such polymerization is feasible because both amphotericin B and borate are bifunctional species capable of forming extended coordination networks. In this model, borate ions act as bridging units that link neighboring amphotericin B molecules through reversible borate ester interactions. The equilibrium governing complex formation is therefore strongly favored at elevated pH values [126,127].

Spectroscopic analysis using UV–visible absorption and circular dichroism (CD) spectroscopy demonstrated that amphotericin B molecules are capable of reversible stacking interactions, leading to dimer formation. These stacking interactions arise primarily from π–π interactions between the extended polyene chromophores of neighboring molecules. Dimerization constants derived from these spectral data were maximal under neutral conditions and decreased under both acidic and alkaline conditions. In alkaline media, the amphotericin B–borate polymer chains are relatively long and extended, exhibiting minimal polyene stacking. In contrast, at neutral pH the polymer chains are shorter and display extensive stacking interactions. Under acidic conditions, where borate complexation does not occur, amphotericin B molecules exhibit an intermediate degree of stacking [128,129,130,131].

Importantly, the distinct and contrasting effects of pH and concentration on borate complexation versus polyene dimerization demonstrate that these two equilibria are mechanistically independent. This decoupling highlights the complexity of amphotericin B self-association behavior and underscores the potential of borate coordination as a strategy to modulate aggregation, solubility, and possibly biological activity of polyene macrolide antibiotics. Understanding these interactions may therefore provide new opportunities for the rational design of improved amphotericin B formulations with enhanced pharmacological properties and reduced toxicity.

Amphotericin B is a potent, broad-spectrum antifungal antibiotic belonging to the polyene macrolide class. For more than six decades, it has remained a cornerstone of antifungal therapy and is still widely regarded as the gold standard for the treatment of severe, life-threatening systemic fungal infections. Its clinical importance persists despite the development of newer antifungal agents [132,133,134].

The primary mechanism of action of amphotericin B involves its high-affinity binding to ergosterol, the principal sterol component of fungal cell membranes. Upon binding, amphotericin B forms transmembrane pores or ion-conducting channels, resulting in increased membrane permeability. This disruption leads to the uncontrolled leakage of essential intracellular ions and metabolites, ultimately causing fungal cell death [135,136,137].

Amphotericin B exhibits activity against a broad range of pathogenic fungi, including most species of *Candida* and *Aspergillus*, as well as the etiological agents of cryptococcosis, histoplasmosis, mucormycosis, and other invasive mycoses. In addition to its antifungal properties, amphotericin B is also employed in the treatment of the protozoal infection visceral leishmaniasis, underscoring its broad antiparasitic potential [138,139,140,141].

Amphotericin B is a potent, broad-spectrum polyene macrolide antibiotic that has remained the gold standard for the treatment of severe, life-threatening systemic fungal infections for more than six decades. Its primary mechanism of action involves high-affinity binding to ergosterol, the dominant sterol component of fungal cell membranes, followed by the formation of transmembrane pores or ion-conducting channels. These pores disrupt membrane integrity, leading to leakage of essential intracellular ions (predominantly K^+^, but also Ca^2+^ and Mg^2+^) and small metabolites, culminating in fungal cell death. In addition to its antifungal activity, amphotericin B is clinically effective against protozoan parasites such as *Leishmania* spp., highlighting its broad antimicrobial spectrum [142,143,144].

**Figure 5:**
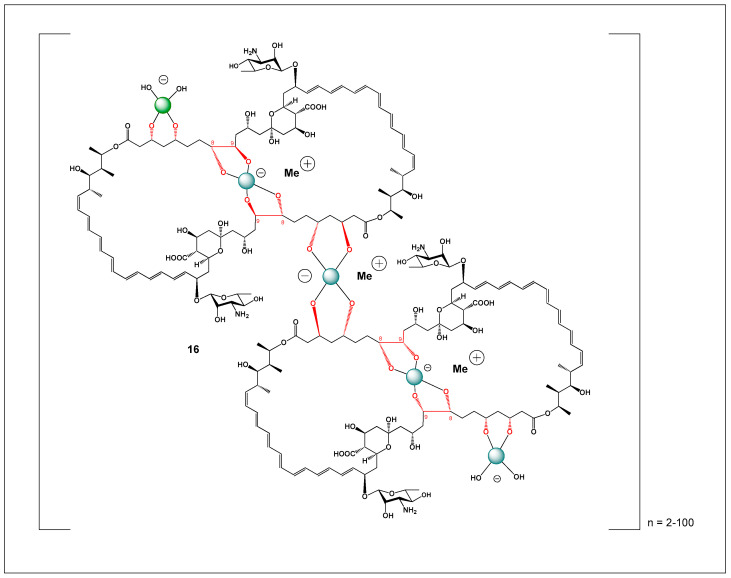
The amphotericin B–borate complex (AmB–borate) was characterized by composition, molecular weight, and spectroscopic properties. Fluorescence and UV–visible analyses indicated a boron-to-polyene molar ratio of approximately 2.65 in neutral aqueous solution (pH 6.3). Differential ultrafiltration showed that at pH 7.0 the complex has an average molecular weight of ~100,000 Da, corresponding to polymeric assemblies of roughly 100 amphotericin B molecules. UV–visible spectra revealed that aggregation of the AmB–borate complex occurs at concentrations about 200-fold higher than for free amphotericin B, indicating strong suppression of polyene aggregation. These results suggest that the complex exists predominantly as extended polymeric chains composed of alternating amphotericin B and borate units, in which borate and tetraborate anions reversibly bridge vicinal cis-diol groups of neighboring amphotericin B molecules.

Despite its exceptional efficacy, the clinical use of amphotericin B is severely limited by its poor aqueous solubility, strong tendency to self-aggregate, and dose-limiting toxicity—most notably nephrotoxicity. These drawbacks have motivated extensive research into formulation strategies aimed at modulating its physicochemical and biological properties. One such strategy involves complexation with borate ions. Amphotericin B contains multiple hydroxyl groups capable of coordinating boric acid or borate anions, leading to the formation of borate–amphotericin B complexes (**16**) (see Figure 5) [126,127,128,145,146]. These complexes exhibit significantly enhanced aqueous solubility and reduced aggregation relative to the parent compound. Importantly, borate complexation alters the aggregation state and supramolecular organization of amphotericin B without abolishing its antifungal activity, thereby offering a potential route to reduced toxicity through controlled membrane interactions.

Mechanistically, the behavior of amphotericin B shares notable parallels with boron-containing ionophore antibiotics such as boromycin, aplasmomycin, borophycin, and hyaboron. These compounds are polyketide-derived macrodiolides that form stable boron-centered complexes through coordination with vicinal diol groups, generating neutral or tetrahedral borate structures. Like amphotericin B, boron-containing ionophore antibiotics disrupt ionic homeostasis by selectively transporting monovalent cations—particularly K^+^—across biological membranes. This ionophoric activity underlies their antibacterial, antifungal, antiparasitic, and cytotoxic effects.

However, a key mechanistic distinction lies in the mode of membrane interaction. Amphotericin B exerts its activity primarily through sterol-dependent pore formation, whereas boron-containing ionophore antibiotics act as mobile carriers or channels that shuttle ions across lipid bilayers independent of sterol binding. Boron plays a central structural and functional role in these ionophores by stabilizing macrocyclic conformations, enhancing membrane permeability, and enabling selective cation coordination. In this context, borate complexation of amphotericin B can be viewed as a convergence between polyene pore-forming antibiotics and boron-mediated ionophoric systems, where boron coordination modulates aggregation, ion transport behavior, and biological selectivity.

From a formulation and medicinal chemistry perspective, these observations suggest that boron coordination represents a powerful and underexplored strategy for tuning the balance between efficacy and toxicity in membrane-active antibiotics. By influencing aggregation state, ion transport dynamics, and membrane affinity, borate complexation may enable the development of next-generation amphotericin B formulations and inspire new hybrid antibiotic designs that bridge polyene and boron-ionophore pharmacology.

## 5. Nystatin and Its Boron Complexes

Nystatin (17), produced by *Streptomyces noursei*, was the first polyene macrolide antifungal antibiotic to be discovered [147,148,149]. Its antifungal activity arises from high-affinity binding to sterols in fungal cell membranes—primarily ergosterol—which leads to increased membrane permeability and leakage of intracellular components, ultimately resulting in cell death [150,151,152]. The resulting disruption of ionic gradients and loss of essential metabolites rapidly compromises cellular integrity and metabolic activity. Structurally, nystatin contains an extended conjugated polyene chain and a polyhydroxylated macrolide ring that together facilitate selective interaction with sterol-containing membranes. In contrast to many other antibiotics, nystatin exhibits no antibacterial activity and is therefore used almost exclusively for the topical treatment of *Candida* infections, including oral, gastrointestinal, and mucocutaneous candidiasis [153,154,155].

From a biosynthetic perspective, the production of nystatin, like that of other polyene macrolides, is strongly influenced by carbon source availability and metabolic regulation in *Streptomyces*. Species-specific variation exists with respect to both cellular growth and secondary metabolite production [156,157,158]. Glucose, while often an excellent substrate for biomass accumulation, has been shown to repress the biosynthesis of numerous antibiotics, including polyene macrolides [159,160,161]. This phenomenon reflects the metabolic prioritization of primary growth processes over energetically expensive secondary metabolism. Indeed, at least 21 secondary metabolites have been reported to be subject to glucose-mediated repression during fermentation [162,163,164]. Other carbohydrates—such as glycerol, maltose, mannose, sucrose, and xylose—can similarly interfere with secondary metabolism, although the magnitude and direction of these effects vary among strains and compounds [165,166].

The mechanism underlying catabolite repression in *Streptomyces* differs from classical carbon catabolite repression observed in other bacteria and remains incompletely understood. Current evidence suggests a unique regulatory system that controls the rate of carbon utilization rather than acting solely through a single global transcriptional regulator [167,168]. This regulatory complexity reflects the intricate metabolic networks governing secondary metabolite production in filamentous actinomycetes. Such regulatory pathways integrate environmental signals, nutrient availability, and cellular developmental states. This regulatory complexity has important implications not only for nystatin production yields but also for the intracellular availability of highly oxygenated polyene macrolides capable of forming supramolecular assemblies or metal–metalloid complexes, including potential interactions with borate species under appropriate environmental or formulation conditions.

Nystatin is a polyene macrolide antifungal antibiotic closely related in structure and mechanism of action to amphotericin B. Like these compounds, nystatin contains an extended conjugated polyene region and a polyhydroxylated macrolide backbone capable of interacting with sterols, primarily ergosterol, in fungal membranes. These structural elements enable the formation of membrane-associated aggregates that participate in pore formation and membrane destabilization. However, in contrast to amphotericin B, nystatin is characterized by extremely poor systemic absorption, which confines its pharmacological activity largely to local or luminal environments and substantially reduces systemic toxicity. This pharmacokinetic property has made nystatin particularly valuable for topical and gastrointestinal antifungal therapy while limiting its application in systemic infections.

From a chemical perspective, nystatin also possesses multiple vicinal and non-vicinal hydroxyl groups within its macrolide framework, theoretically enabling coordination with boric acid or borate anions via reversible diol–borate complexation [169,170]. This structural feature places nystatin within the same boron-binding chemical space as amphotericin B. Nevertheless, unlike amphotericin B, for which borate complexation has been experimentally demonstrated to reduce aggregation, enhance solubility, and modulate toxicity, boron–nystatin complexes have not been extensively developed or exploited clinically.

This difference is largely attributable to pharmacological necessity rather than chemical incompatibility. Amphotericin B suffers from pronounced aggregation in aqueous media and severe dose-limiting nephrotoxicity, which has driven extensive formulation research, including borate complexation, lipid carriers, and nanoparticle systems. In contrast, nystatin’s intrinsic lack of systemic exposure minimizes the clinical relevance of aggregation-related toxicity, reducing the incentive to pursue boron-mediated formulation strategies. As a result, nystatin serves as an instructive counterexample within the polyene family: a molecule chemically capable of borate coordination, yet pharmacologically exempt from the need for boron-based toxicity modulation [169,170].

Mechanistically, this comparison highlights an important principle of boron–polyene chemistry: borate complexation does not fundamentally alter the sterol-binding mechanism shared by polyene macrolides, but rather modulates supramolecular properties such as aggregation state, solubility, membrane interaction kinetics, and ion channel formation. In amphotericin B, these effects translate into altered biological activity and toxicity profiles, whereas in nystatin they remain largely latent due to the drug’s localized mode of action.

Taken together, nystatin underscores the dual nature of boron chemistry in polyene antibiotics—structurally permissive yet biologically context-dependent—and illustrates how boron coordination becomes pharmacologically meaningful only when aggregation-driven toxicity and systemic exposure are limiting factors.

**Figure 6:**
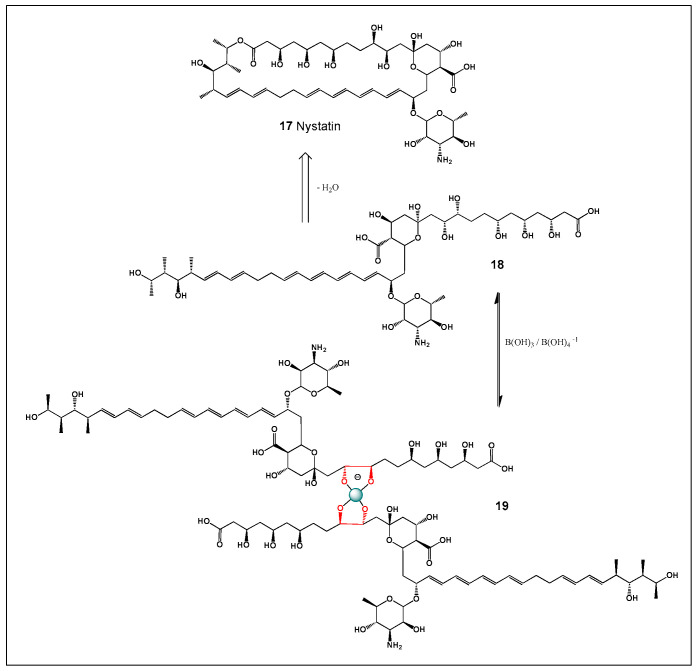
Proposed biosynthetic pathway of nystatin and formation of its boron complex. Nystatin contains multiple vicinal 1,2-diol motifs within its polyol region, which serve as effective coordination sites for boric acid and borate anions. Similarly to other highly oxygenated polyene macrolides, these cis-diol groups can reversibly form cyclic borate esters, yielding neutral or tetrahedral boron–polyene complexes depending on solution pH and the prevailing boron species. The formation of nystatin–borate complexes is therefore chemically plausible and consistent with well-established boron–diol coordination chemistry. Boron complexation may alter the aggregation behavior of nystatin, modulate its interactions with fungal membranes, and enhance its aqueous solubility and local bioavailability. As a result, borate complexes of nystatin may exhibit improved antifungal efficacy compared with the parent antibiotic, particularly under physiological or formulation-relevant conditions.

Structurally, nystatin contains multiple vicinal 1,2-diol motifs within its polyol region, which provide suitable coordination sites for boric acid and borate anions. As with other highly oxygenated polyene macrolides, these cis-diol groups can reversibly form cyclic borate esters, resulting in neutral or tetrahedral boron–polyene complexes depending on pH and boron speciation. The formation of nystatin–borate complexes is therefore chemically plausible and consistent with well-established boron–diol coordination chemistry. Such complexation may influence nystatin’s aggregation state, membrane interactions, and local bioavailability, potentially modulating both antifungal potency and toxicity, analogous to effects observed for amphotericin B–borate system. Nystatin is formed from the polyketide precursor (**18**) by ester bond formation, and the boron complex (**19**) is formed by the interaction of the precursor (**18**) and boric acid or boron anion (Figure 6).

## 6. Fungichromin and Its Boron Complexes

An endophytic strain, *Streptomyces* sp. WP-1, was isolated from surface-sterilized bark tissues of *Pinus dabeshanensis* [171,172,173,174]. Endophytic actinomycetes are well known as prolific producers of bioactive secondary metabolites that contribute to host defense against microbial pathogens. The strain exhibited pronounced antifungal activity against a broad spectrum of phytopathogenic and opportunistic fungi, including *Fusarium oxysporum*, *Rhizoctonia solani*, *Phytophthora infestans*, and *Candida albicans*. Such activity suggests that the microorganism may play a protective ecological role within plant tissues by suppressing pathogenic fungi. Phylogenetic analysis based on 16S rRNA gene sequencing confirmed that strain WP-1 belongs to the genus *Streptomyces*. Members of this genus are widely recognized for their capacity to produce structurally diverse polyketide antibiotics and other biologically active natural products.

High-performance liquid chromatography (HPLC) of the WP-1 culture extract enabled isolation of the major antifungal metabolite, which was identified as fungichromin (FC, 20), a methylpentaene macrolide antibiotic. Structural characterization confirmed that the compound belongs to the family of polyene macrolide antibiotics containing an extended conjugated polyene system and a polyhydroxylated macrolactone ring. In vitro bioassays demonstrated that fungichromin potently inhibited both mycelial growth and conidial germination of *F. oxysporum*, confirming its strong antifungal efficacy. These findings highlight the potential of endophytic *Streptomyces* species as promising sources of antifungal agents for both medical and agricultural applications.

**Figure 7:**
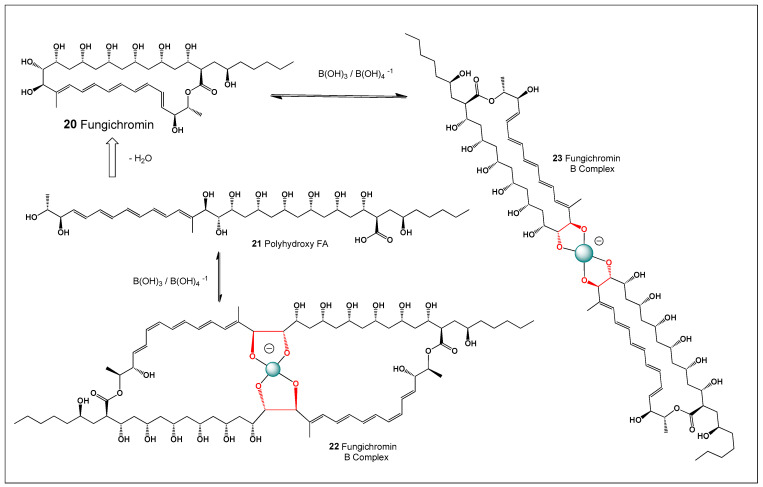
Putative biosynthesis of fungichromin from a polyhydroxy polyketide precursor and formation of boron-coordinated complexes. Fungichromin, a polyene macrolide antifungal antibiotic produced by *Streptomyces* species (e.g., *S. pentaticus* and *S. padanus*), is biosynthesized through a modular polyketide synthase (PKS) pathway that generates a polyhydroxylated macrolactone containing multiple vicinal diol motifs. These diol groups enable reversible coordination with boric acid or borate anions, potentially forming fungichromin–borate complexes. Fungichromin acts by binding ergosterol in fungal membranes, leading to pore formation, membrane permeabilization, and leakage of intracellular ions. Clinically it has been used for the treatment of vaginal infections and is also applied as an agricultural biofungicide. Boron coordination may influence fungichromin aggregation, solubility, and membrane interactions, similar to amphotericin B–borate systems.

The figure illustrates the proposed biosynthetic pathway of fungichromin via a modular polyketide synthase (PKS) system, leading to formation of the polyene macrolactone scaffold. Such PKS systems assemble complex polyketide structures through sequential condensation of simple acyl-CoA precursors followed by extensive tailoring reactions. In addition, a putative pathway for boron complex formation is shown (Figure 7), in which vicinal 1,2-diol motifs within the polyol region of fungichromin reversibly coordinate boric acid or borate anions. These polyhydroxylated regions provide suitable binding sites for boron through cyclic borate ester formation. Such post-biosynthetic complexation may modulate molecular aggregation, membrane interactions, and biological activity, analogous to borate complexes observed for other polyene macrolide antibiotics [174,175,176]. The possibility of boron coordination therefore adds an additional layer of chemical complexity that may influence the pharmacological and physicochemical behavior of fungichromin.

### Comparison of Fungichromin–Borate and Amphotericin B–Borate Complexes

Fungichromin and AmB belong to the same structural class of polyene macrolide antibiotics, sharing a large macrolactone ring, an extended conjugated polyene chain, and a polyhydroxylated region containing vicinal diol motifs. These diol-rich domains provide chemically defined coordination sites for boric acid and borate anions, enabling reversible boron complex formation in aqueous environments.

In amphotericin B, borate complexation has been shown to occur through coordination of borate species with neighboring hydroxyl groups in the polyol region, leading to the formation of polymeric copolymer chains in which amphotericin B and borate units alternate. This complexation is strongly pH-dependent, favored under neutral to alkaline conditions, and results in a marked increase in aqueous solubility, reduced aggregation, and altered molecular conformation. Importantly, borate complexation modulates AmB self-association independently of polyene dimerization, thereby influencing membrane interactions and reducing nonspecific toxicity toward mammalian cells while preserving antifungal activity.

Fungichromin possesses a closely related polyene–polyol architecture and similarly contains multiple cis-vicinal hydroxyl groups capable of acting as bidentate ligands for boron coordination. By analogy with amphotericin B, fungichromin is therefore expected to form fungichromin–borate complexes through borate bridging between adjacent diols, yielding extended but dynamically reversible assemblies. Such complexation is likely to decrease polyene aggregation in aqueous media, enhance solubility, and modulate interactions with sterol-containing membranes.

A key mechanistic distinction lies in their biological targets and membrane selectivity. Amphotericin B preferentially binds ergosterol over cholesterol, forming transmembrane pores that induce ion leakage. Borate complexation reduces AmB aggregation and pore-forming activity in cholesterol-rich membranes, thereby mitigating nephrotoxicity. Fungichromin, while also disrupting fungal membranes, displays a narrower antifungal spectrum and reduced mammalian toxicity. Borate complexation of fungichromin may therefore further bias membrane interactions toward fungal sterols, enhancing antifungal selectivity while limiting host cell damage.

In both cases, boron acts not as a covalent structural element but as a dynamic coordination center, reversibly linking polyene macrolide units through diol–borate interactions. This shared mechanism underscores a generalizable strategy by which boron coordination modulates the physicochemical and biological properties of polyene macrolide antibiotics. The fungichromin–borate system thus represents a functional analog of the amphotericin B–borate complex and supports the broader concept that boron can fine-tune antibiotic efficacy and toxicity through supramolecular assembly rather than permanent chemical modification.

## 7. Sorangicin A and Its Boron Complex

**Figure 8:**
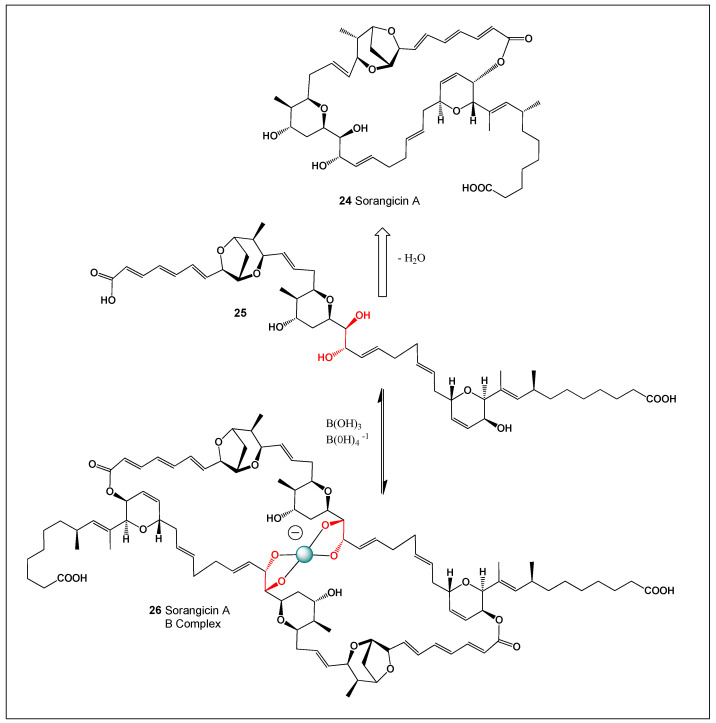
Sorangicin A contains a vicinal 1,2-diol motif within its highly oxygenated macrolide framework, providing an appropriate coordination site for boric acid or borate anions. As a result, Sorangicin A is capable of forming reversible boron complexes through cyclic borate ester formation. These Sorangicin A–boron complexes appear to display enhanced selectivity toward Gram-positive bacteria, likely due to boron-mediated modulation of molecular conformation, membrane permeability, and RNA polymerase binding efficiency.

Sorangicin A (SorA, **24**, Figure 8) is a highly potent and structurally complex macrolide antibiotic produced by Myxobacteria. It exerts its antibacterial activity by selectively inhibiting bacterial RNA polymerase (RNAP), in a manner mechanistically analogous to rifampicin, thereby blocking transcription initiation. Owing to this mode of action, SorA displays broad-spectrum antibacterial activity, including efficacy against difficult-to-treat intracellular pathogens such as *Chlamydia* species [177,178,179].

SorA is particularly active against Gram-positive bacteria, including mycobacteria, with minimum inhibitory concentration (MIC) values as low as 0.01 µg/mL. At higher concentrations (MIC ≈ 3 µg/mL), inhibitory effects extend to certain Gram-negative bacteria. In contrast, yeasts and filamentous fungi are completely resistant, consistent with the compound’s specificity for eubacterial RNA polymerase [180,181,182].

Mechanistically, SorA acts as a highly specific inhibitor of eubacterial RNAP and is effective only when administered prior to the initiation of RNA polymerization, indicating that it interferes with early transcriptional events rather than elongation. Its unique molecular architecture and potent transcriptional inhibition make SorA an attractive lead compound for the development of next-generation antibacterial agents, particularly in the context of rising resistance to existing RNAP-targeting antibiotics.

SorA contains multiple oxygenated functional groups within its macrolide framework, including at least one vicinal 1,2-diol motif, which provides a chemically plausible coordination site for boric acid or borate anions. As established for other highly oxygenated polyketide antibiotics, such *cis*-diol units can reversibly form cyclic borate esters, yielding tetrahedral boron complex (**26**) from the precursor (**25**) depending on pH and boron speciation. The formation of SorA–borate complexes is therefore consistent with well-documented boron–diol coordination chemistry observed in polyketide antibiotics such as boromycin, aplasmomycin, amphotericin B, fungichromin, and nystatin [49,50,183].

Functionally, boron complexation is expected to modulate the physicochemical and biological properties of sorangicin A, including its aggregation state, membrane permeability, and target selectivity. Notably, preliminary observations suggest that Sorangicin A–borate complexes exhibit enhanced selectivity toward Gram-positive bacteria, while maintaining potent inhibition of bacterial RNA polymerase (RNAP). This selectivity may arise from altered uptake across the thick peptidoglycan layer of Gram-positive organisms or from changes in intracellular distribution and binding kinetics of the antibiotic–boron complex.

### 7.1. Comparison of SorA with Rifampicin: Binding Site and Resistance Profile

SorA and rifampicin share a common molecular target, the β-subunit of eubacterial RNA polymerase, and both inhibit transcription by blocking the formation of the first phosphodiester bond during RNA synthesis. However, despite this functional similarity, their binding modes differ significantly at the molecular level. Rifampicin binds deeply within the RNAP exit channel and interacts primarily through hydrophobic and π–π interactions, making its activity highly sensitive to point mutations in the rpoB gene. As a result, rifampicin resistance arises rapidly and is widespread among pathogenic bacteria [184,185].

In contrast, SorA binds RNAP through a distinct, partially overlapping but conformationally flexible binding site, engaging additional hydrogen-bonding and macrolide-specific interactions. This difference confers a reduced cross-resistance with rifampicin, and several rifampicin-resistant RNAP mutants remain susceptible to sorangicin A. The presence of a boron-complexed form of sorangicin A may further stabilize RNAP binding or alter interaction geometry, potentially lowering the probability of resistance development.

Taken together, the combination of RNA polymerase inhibition, boron-diol complexation capability, enhanced Gram-positive selectivity, and a resistance profile distinct from rifampicin positions SorA and its boron complexes as promising candidates for the development of next-generation transcription-targeting antibiotics.

**Figure 9:**
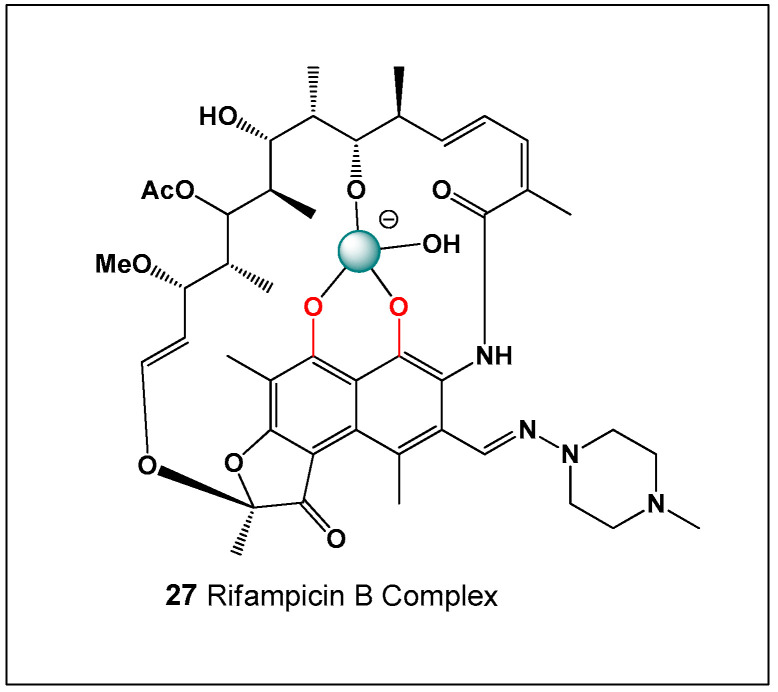
Chemical structure of rifamycin, a member of the ansamycin class of antibiotics. Although rifamycin is not a polyene macrolide, it exhibits broad-spectrum antibacterial activity by inhibiting DNA-dependent RNA polymerase. The molecule contains multiple hydroxyl groups capable of coordinating boric acid or borate anions, enabling the formation of reversible boron complexes that may influence its physicochemical properties and biological activity. Rifamycin is characterized by a 1,3-diol system during the formation of the boron complex. Details are described in [25].

Rifamycin (**27**) is a bactericidal, broad-spectrum antibiotic belonging to the ansamycin class. Its antibacterial activity arises from high-affinity binding to DNA-dependent RNA polymerase, thereby inhibiting RNA synthesis at the transcriptional level [186,187,188,189]. By blocking the elongation of nascent RNA chains, rifamycin effectively suppresses bacterial gene expression and ultimately leads to cell death. Structurally, rifamycin contains three conformationally aligned hydroxyl groups that create a favorable coordination environment for boric acid or borate anions. These hydroxyl functionalities enable the formation of reversible rifamycin–boron complexes through borate ester interactions. The polyoxygenated ansa bridge of rifamycin further stabilizes such coordination through intramolecular hydrogen bonding and spatial alignment of donor atoms. Such complexation has the potential to influence molecular conformation, target engagement, and pharmacological behavior [190,191,192]. In particular, boron coordination may alter the orientation of key functional groups involved in RNA polymerase binding, potentially modulating antibiotic potency. Furthermore, reversible borate ester formation could affect the solubility, aggregation state, and environmental persistence of rifamycin under certain conditions. These chemical interactions highlight the broader relevance of boron coordination chemistry in shaping the biological activity of polyhydroxylated natural product antibiotics. The chemical structure of rifamycin is shown in Figure 9.

### 7.2. Comparative Analysis of Rifamycin–Borate and Sorangicin A–Borate Complexes

Rifamycin and sorangicin A are structurally distinct macrolide antibiotics that share a common molecular target—DNA-dependent RNA polymerase (RNAP)—yet differ markedly in their binding modes, resistance profiles, and physicochemical properties. Both molecules possess vicinal or conformationally aligned hydroxyl groups, enabling reversible complexation with boric acid or borate anions, a feature that may modulate their biological activity and selectivity.

#### 7.2.1. Binding Site Overlap and RNAP Inhibition

Rifamycin binds within the RNAP β-subunit rifampicin-binding pocket, sterically blocking the elongation of nascent RNA chains after the formation of the first few phosphodiester bonds. This binding site is well-defined and conserved, but also highly susceptible to resistance-conferring mutations (e.g., in the rpoB gene).

Sorangicin A occupies an overlapping but non-identical binding region on RNAP. While it also inhibits transcription initiation, sorangicin A interacts with additional residues outside the canonical rifampicin pocket, engaging a broader surface area of RNAP. This expanded interaction network explains why sorangicin A retains activity against many rifampicin-resistant bacterial strains.

Formation of borate complexes with either antibiotic may subtly influence RNAP binding by: (i) stabilizing specific conformations of the macrolide scaffold; (ii) altering hydrogen-bonding patterns within the binding pocket; (iii) modulating steric complementarity at the protein–ligand interface.

#### 7.2.2. Resistance Profiles

Resistance to rifamycin arises rapidly through single-point mutations in RNAP that disrupt drug binding without compromising enzyme function. Because rifamycin binds in a narrow, highly specific pocket, even minor structural perturbations drastically reduce affinity.

In contrast, sorangicin A demonstrates a lower frequency of resistance development, as mutations conferring resistance often require multiple or structurally destabilizing changes in RNAP. This difference is particularly relevant in the context of boron complexation: sorangicin A–borate complexes, by modifying molecular flexibility or spatial orientation, may further reduce susceptibility to resistance by maintaining productive binding even in mutated RNAP variants.

#### 7.2.3. Selectivity and Spectrum of Activity

Rifamycin exhibits broad-spectrum antibacterial activity but is limited by resistance emergence and dose-limiting toxicity. Borate complexation of rifamycin—mediated by its clustered hydroxyl groups—may influence aggregation, solubility, and tissue distribution, potentially altering its therapeutic window.

Sorangicin A, by contrast, shows enhanced selectivity toward Gram-positive bacteria, with limited activity against Gram-negative organisms unless higher concentrations are used. The presence of a 1,2-diol motif in sorangicin A supports the formation of defined boron complexes, which may: (i) favor conformations that better recognize Gram-positive RNAP; (ii) reduce nonspecific interactions; (iii) enhance intracellular retention in Gram-positive cells.

Thus, sorangicin A–borate complexes appear inherently more selective RNAP inhibitors, combining structural adaptability with a resistance-resilient binding mode.

#### 7.2.4. Conceptual Implications

Together, these observations suggest that boron coordination chemistry introduces an additional layer of functional modulation for RNAP-targeting antibiotics. While rifamycin–borate complex (**27**) may primarily affect physicochemical properties, sorangicin A–borate complexes may directly enhance selectivity and resistance robustness through structural and mechanistic synergy.

This comparison underscores the broader concept of “boron-assisted antibiotic tuning”, in which reversible boron–diol interactions fine-tune target engagement, resistance profiles, and biological specificity.

## 8. Tetracycline Antibiotics and Their Boron Complexes

**Figure 10:**
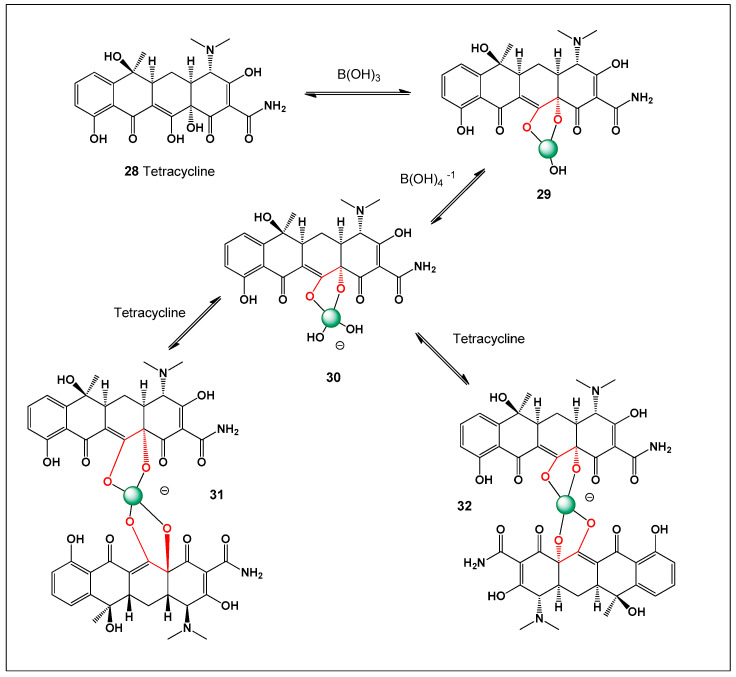
The interaction of tetracyclines with boric acid and borate anions, leading to the formation of coordination complexes, has been extensively studied. While these complexes are well characterized chemically, their potential implications for pharmacological behavior and biological activity continue to attract interest and warrant further investigation.

**Figure 11:**
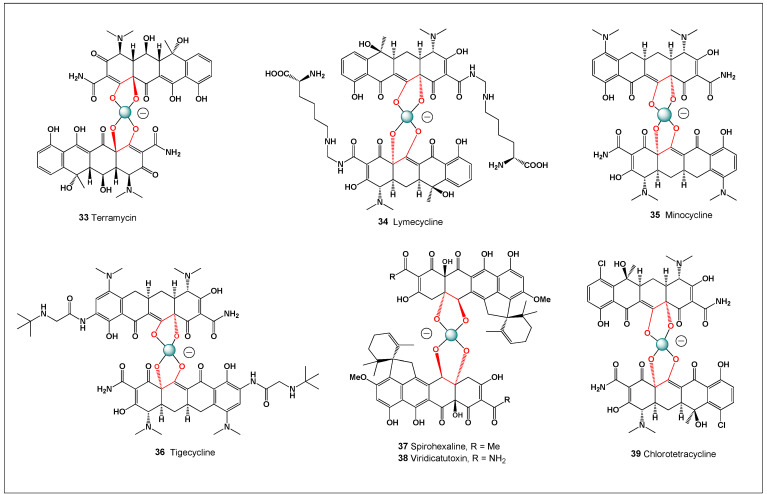
The formation of stable yet reversible complexes between tetracyclines and boric acid or borate anions is enabled by the presence of vicinal diol moieties in the tetracycline scaffold. Such boron–tetracycline complexes are of growing interest in medicinal chemistry and pharmacology, as boron coordination may influence antibacterial activity, toxicity, and interactions with biological targets.

Tetracycline antibiotics (28–39, Figure 10 and Figure 11) represent one of the major classes of antimicrobial agents used extensively in both human and veterinary medicine. They were discovered in the 1940s and rapidly gained widespread clinical application due to their broad-spectrum antibacterial activity [193,194,195,196]. Tetracyclines are effective against a wide range of Gram-positive and Gram-negative bacteria, as well as certain atypical pathogens including *Chlamydia*, *Mycoplasma*, and *Rickettsia*. Their versatility and relatively low cost have contributed to their long-standing importance in clinical therapy. Despite decades of use, tetracyclines continue to serve as valuable scaffolds for the development of new antibacterial agents designed to overcome emerging resistance.

Naturally occurring tetracyclines are highly oxygenated type II polyketides characterized by a linearly fused tetracyclic backbone composed of four rings, conventionally designated A, B, C, and D. These compounds are biosynthesized primarily by *Streptomyces aureofaciens* and *Streptomyces rimosus*. Additional naturally occurring tetracyclines were subsequently identified, including tetracycline itself from *S. aureofaciens*, *S. rimosus*, and *S. viridofaciens*, as well as dimethylchlortetracycline from *S. aureofaciens*. The molecular framework of tetracyclines contains multiple hydroxyl, keto, and amide functional groups that contribute to their strong metal-chelating properties. Advances in medicinal chemistry later enabled the development of semisynthetic tetracyclines, such as methacycline, doxycycline, and minocycline, which exhibit improved pharmacokinetic and antibacterial properties [197,198,199]. These modifications have expanded the therapeutic usefulness of the tetracycline class while improving stability, absorption, and tissue penetration.

Tetracyclines exert their antibacterial activity by inhibiting protein synthesis. Specifically, they bind to the 16S rRNA of the 30S bacterial ribosomal subunit, thereby preventing the accommodation of aminoacyl-tRNA into the ribosomal acceptor (A) site. This interaction effectively halts peptide chain elongation and suppresses bacterial growth [200,201,202]. At the molecular level, tetracyclines establish sequence-independent interactions with the sugar–phosphate backbone of rRNA within the primary binding pocket located between helices h31 and h34 of the 16S rRNA [201,202,203]. The binding process often involves coordination with divalent metal ions such as Mg^2+^, which help stabilize the antibiotic–ribosome complex. Because tetracyclines possess multiple oxygen-containing functional groups, they also have the potential to form reversible complexes with boron species through diol and keto–enol coordination motifs. Such interactions may influence the conformational flexibility and physicochemical behavior of tetracycline molecules under certain environmental or biochemical conditions. These properties make tetracyclines particularly relevant when considering the broader role of boron coordination in modulating the activity of polyoxygenated natural product antibiotics.

Tetracycline and its clinically important derivatives—chlortetracycline, oxytetracycline, methacycline, doxycycline, minocycline, tigecycline, omadacycline, and eravacycline—contain vicinal diol functional groups at the C-12 and C-12a positions. These *cis*-1,2-diol motifs readily interact with boric acid to form stable boron complexes. This property has been successfully exploited in analytical chemistry, particularly for the separation and quantification of tetracycline antibiotics using capillary zone electrophoresis coupled with fast cyclic voltammetric detection. Optimization of pH conditions and complexation with boric acid or sodium tetraborate enabled efficient resolution and accurate quantification of tetracycline, chlortetracycline, and oxytetracycline, demonstrating the strong and selective affinity of these antibiotics for boron-containing species [204,205,206,207].

Over geological timescales, oceanic environments have developed exceptionally rich and unique microbial diversity distributed throughout seawater, marine sediments, and marine-associated organisms [208,209,210]. In contrast to terrestrial ecosystems traditionally explored in natural product research, marine microbial habitats provide distinct physicochemical conditions that favor the biosynthesis of structurally unusual and highly bioactive secondary metabolites. Among marine microorganisms, actinomycetes represent a particularly prolific source of natural products, producing a wide array of antibiotics, toxins, and other pharmacologically potent compounds, including tetracycline-related molecules [211,212,213,214].

Polyketide natural products constitute a dominant class of secondary metabolites produced by microorganisms and account for a substantial proportion of known bioactive compounds. These molecules arise primarily from the stepwise condensation of short-chain fatty acid precursors catalyzed by polyketide synthases, followed by extensive post-assembly modifications such as oxidation, reduction, and hydroxylation [215,216]. These biosynthetic transformations generate remarkable structural diversity and confer a broad spectrum of biological activities. Notably, several structurally unique polyketide macrolides (compounds **33**–**39**, Figure 11), featuring L-rhodinose residues, spiroketal motifs, and an unusual continuous array of hydroxyl groups within the macrolide ring, were recently isolated from the marine Actinomycete *Micromonospora* sp. FIMYZ51, highlighting the chemical novelty accessible from marine-derived microorganisms [217,218,219].

### Biological Consequences of Tetracycline–Boron Complexation: Antibiotic Enhancement Versus Toxicity

The ability of tetracycline antibiotics to form stable complexes with boron through their vicinal *cis*-1,2-diol moieties has implications that extend beyond analytical separation and into biological activity. Boron complexation may influence antibiotic behavior at multiple levels, including molecular stability, membrane transport, target binding, and off-target interactions. On one hand, the formation of neutral or tetrahedral borate complexes can enhance antibiotic performance by modulating physicochemical properties such as solubility, lipophilicity, and resistance to enzymatic degradation. These effects may facilitate improved penetration across bacterial membranes or alter intracellular distribution, potentially increasing antibacterial efficacy under specific physiological conditions [204,205,206,207].

Conversely, boron complexation can also shift tetracyclines toward toxicological outcomes. Boron–tetracycline complexes may perturb metal ion homeostasis by competing with physiologically essential divalent cations such as Mg^2+^ and Ca^2+^, which are crucial for ribosomal binding and cellular signaling. Such competition may disrupt normal ribosomal function not only in bacteria but also in host cells, thereby contributing to cytotoxic or off-target effects. In addition, boron complexes may alter redox behavior or promote unintended interactions with nucleic acids, membranes, or enzymes, particularly under conditions of elevated boron availability.

From an ecological and evolutionary perspective, environments enriched in boron—such as marine systems or boron-rich soils—may favor the biosynthesis or stabilization of boron–antibiotic complexes, thereby influencing microbial competition and chemical defense strategies [220,221,222,223,224,225]. In this context, tetracycline–boron interactions exemplify boron’s dual role as both a functional enhancer of antimicrobial activity and a potential mediator of toxicity. This duality underscores the importance of considering boron not merely as a passive environmental element but as an active chemical participant capable of modulating the biological effects of antibiotic molecules.

## 9. Toxins and Their Boron Complexes

Toxins are natural organic compounds—collectively referred to as poisons—produced primarily by microorganisms (including bacteria and fungal endophytes), as well as by plants and animals [226,227,228]. These substances often serve ecological functions such as defense against predators, inhibition of competing organisms, or facilitation of predation. In many cases, organisms that do not biosynthesize these compounds can accumulate, transform, or partially metabolize them through dietary or symbiotic interactions. Such trophic transfer mechanisms are particularly well documented in marine ecosystems, where toxins produced by microorganisms may accumulate in higher organisms through complex food-web interactions. The term toxin was first introduced into scientific literature by the German organic chemist Ludwig Brieger [229], who studied putrefactive alkaloids formed in animal tissues under bacterial activity in the late nineteenth century. Brieger’s work laid the foundation for the scientific study of biologically produced toxic compounds and their chemical characterization. Since then, the concept of toxins has expanded to encompass a wide range of structurally diverse natural products with potent biological activity. Many toxins possess highly oxygenated frameworks that enable interactions with biological macromolecules such as ion channels, enzymes, or ribosomal components. These structural characteristics often overlap with those found in antibiotic natural products, illustrating the fine line between toxic and therapeutic biological activity that is explored throughout this review.

## 10. Tetrodotoxins and Their Boron Complexes

One of the most potent naturally occurring toxins is tetrodotoxin (TTX, 40), the principal toxic metabolite found in puffer fish (family Tetraodontidae), commonly known as fugu. Tetrodotoxin exhibits an exceptionally strong neuroparalytic effect and is estimated to be several hundred times more toxic than potassium cyanide [230,231,232]. Even very small quantities of the toxin can cause rapid onset of neurological symptoms, including numbness, paralysis, and respiratory failure. In Japan, despite strict culinary regulations, dozens of poisoning cases are still reported annually. The preparation of fugu is therefore restricted to specially licensed chefs trained to remove the toxin-containing organs. Although TTX was first isolated in 1909 by Yoshizumi Tahara from puffer fish ovaries, the toxicity of fugu had been recognized long before its chemical characterization [233,234,235].

At the molecular level, TTX acts as a highly selective blocker of voltage-gated sodium (Na_v_) channels. It binds to the outer vestibule of the sodium channel pore, interacting with the selectivity filter and physically preventing sodium ion flux without affecting channel gating kinetics. This exquisite selectivity underlies its profound neurotoxicity. By blocking sodium ion conductance in nerve and muscle cells, TTX effectively prevents the propagation of action potentials required for normal neuromuscular function. More recently, TTX-resistant sodium channel isoforms have been identified in mammalian sensory neurons, where they play key roles in nociception and pain signaling. These discoveries have stimulated interest in TTX and related molecules as potential pharmacological tools for studying neuronal signaling pathways. Notably, despite decades of research, no effective antidote for TTX poisoning is currently available [236,237,238].

Beyond puffer fish, TTX has been detected in a wide array of marine and terrestrial organisms, including crabs, starfish, gastropods, blue-ringed octopuses, amphibians, and even parasitic flatworms [239,240,241]. Importantly, accumulating evidence indicates that TTX is not synthesized by higher organisms themselves but originates from symbiotic or free-living bacteria. TTX-producing strains have been isolated from genera such as *Vibrio*, *Pseudomonas*, *Bacillus*, *Aeromonas*, *Alteromonas*, *Moraxella*, *Flavobacterium*, and others [242,243,244]. These bacteria colonize tissues such as the intestine, liver, skin, and reproductive organs of host animals, allowing the toxin to propagate through marine and terrestrial food webs. Such microbial symbioses are thought to play a key role in maintaining toxin levels within host organisms. Significant concentrations of TTX have also been detected in marine and deep-sea sediments, suggesting a stable environmental reservoir [245,246,247]. The persistence of TTX in sediments further supports the hypothesis that environmental microbial communities serve as long-term sources of the toxin.

An intriguing connection between tetrodotoxin and boron metabolism has recently emerged. Kato and co-workers [248] demonstrated that euryhaline puffer fish (*Takifugu obscurus*) possess an exceptionally efficient renal boron excretion system. Boric acid concentrations in urinary bladder fluid differed by nearly three orders of magnitude between freshwater- and seawater-acclimated fish. Functional studies identified Slc4a11A as an electrogenic boric acid transporter capable of operating as a borate uniporter, boric acid–hydroxide cotransporter, or proton exchanger. This transporter appears to play a central role in boron homeostasis in marine fish and may create physiological conditions favorable for boron–toxin interactions [249]. Such physiological regulation of boron may influence the chemical environment in which tetrodotoxin and related metabolites exist within marine organisms. Because TTX contains multiple hydroxyl groups capable of interacting with boron species, reversible borate complex formation may occur under certain biological conditions. These interactions could potentially affect toxin stability, transport, or bioaccumulation in marine organisms. Consequently, the relationship between boron metabolism and TTX chemistry represents an intriguing area for future investigation in marine chemical ecology.

**Figure 12:**
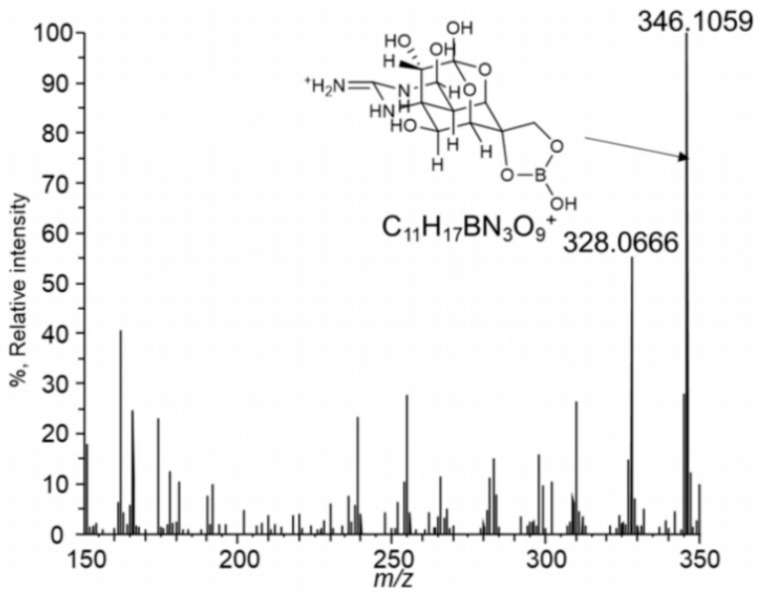
LC–HRMS product ion spectra of Tetrodotoxin (TTX) derived from the precursor ion *m/z* 320.1088. The fragmentation pattern reveals characteristic product ions corresponding to cleavage of the highly oxygenated cage-like framework of TTX. These diagnostic fragments provide structural confirmation of tetrodotoxin and support its identification in complex biological or environmental samples using high-resolution mass spectrometry.

The collision energies were set at 30 eV. Details of the isolation and determination of the structure of the toxin and its boron complex are described in the work [250]. The analytical conditions were optimized to promote reproducible fragmentation of tetrodotoxin and its associated boron-containing species during tandem mass spectrometric analysis. Direct experimental evidence for boron–tetrodotoxin complexation was reported by Daniel Beach and colleagues, who isolated TTX from the tissues of the sea slug *Pleurobranchaea maculata* and the blue mussel *Mytilus edulis* in the form of boron-containing complexes (see Figure 12). Subsequent analytical studies using advanced chromatographic and mass spectrometric techniques, including ion-pair LC–MS/MS and HILIC–MS/MS, further confirmed the existence of TTX–boron associations [250]. These analytical approaches enabled the detection of characteristic mass shifts and fragmentation patterns consistent with borate coordination. The identification of such complexes provides important experimental support for the hypothesis that boron can interact directly with highly oxygenated marine toxins.

The formation of boron complexes with tetrodotoxin is chemically plausible given the dense array of hydroxyl and guanidinium-associated functional groups in the TTX molecule, which can facilitate coordination with boric acid or borate anions. The polyhydroxylated cage-like structure of TTX offers multiple potential binding sites capable of stabilizing cyclic borate ester intermediates. Such complexation may influence toxin stability, solubility, transport, and bioavailability, particularly in marine environments rich in dissolved boron. In aqueous systems, reversible borate ester formation with cis-diol motifs is known to alter molecular conformation and intermolecular interactions. These findings expand the concept of boron coordination beyond antibiotics to include highly potent natural toxins, highlighting boron as a previously underappreciated modulator of both biological activity and environmental fate.

**Figure 13:**
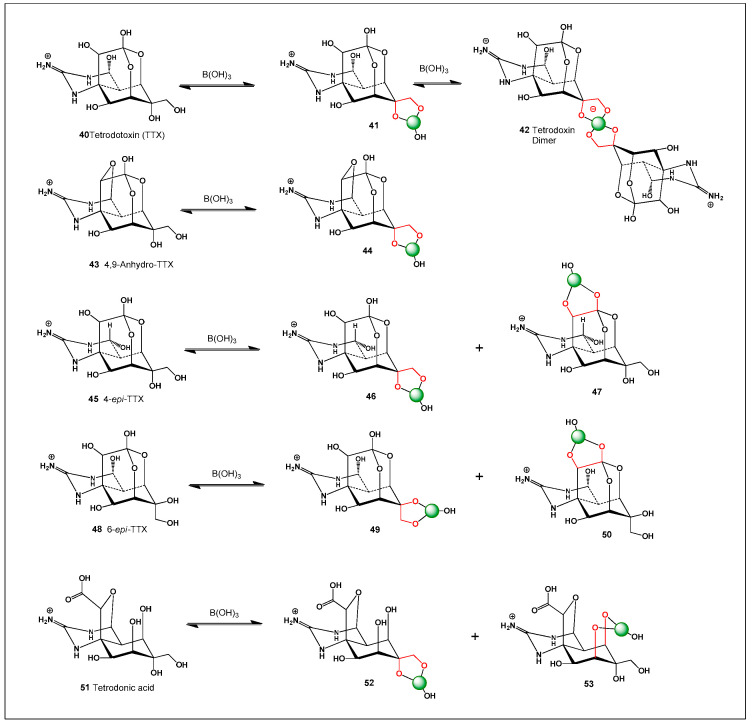
Chemical structures of tetrodotoxin and selected tetrodotoxin derivatives, illustrating the presence of vicinal 1,2-diol motifs capable of coordinating boron species. Under marine conditions, where boron is abundant and exists in pH-dependent equilibrium between boric acid and borate anions, these diol-containing toxins may reversibly form boron complexes. Such complexation could influence toxin stability, solubility, and transport, thereby contributing to bioaccumulation and trophic transfer in marine food webs. Boron coordination may modulate the biological activity of tetrodotoxins by altering their molecular conformation or interaction with voltage-gated sodium channels, although the extent of this effect likely depends on environmental and physiological conditions. Boron complexation may influence the effective toxicity of tetrodotoxins by altering molecular conformation, stability, or bioavailability, although the extent of these effects remains to be fully elucidated.

Based on the principle of mathematical induction—progressing from specific observations to general conclusions—we propose that toxins containing vicinal 1,2-diol moieties, analogous to those present in tetrodotoxin, are inherently capable of forming coordination complexes with boric acid or borate anions (Figure 13). By extrapolating from established examples and applying a principle analogous to mathematical induction, we further suggest that natural toxins bearing vicinal 1,2-diol functionalities—such as tetrodotoxin (40) and derivatives (43, 45, 48, and 51)—are intrinsically capable of forming coordination complexes with boric acid or borate anions. This propensity is particularly relevant in marine environments, where boron is abundant and exists predominantly as boric acid B(OH)_3_ at physiological pH, with increasing contributions of the tetrahedral borate anion [B(OH)_4_]^−^ under slightly alkaline conditions. Under these conditions, reversible formation of cyclic borate esters with cis-diol motifs is chemically favored. Such interactions may contribute to toxin stabilization during trophic transfer within marine food webs. Consequently, boron–toxin complexation may represent an important but largely overlooked factor influencing toxin persistence, distribution, and ecological impact in marine environments.

Considering the high and relatively stable concentration of boron in marine environments, together with its pH-dependent speciation, the ability of vicinal 1,2-diol–containing toxins such as tetrodotoxin (**40**) to form reversible boron complexes (**41**, **42**, **44**, **46**, **47**, **49**, **50**, **52**, and **53**) may have important implications for bioaccumulation and trophic transfer. In marine microorganisms, initial boron–toxin complexation may influence toxin stability, solubility, and cellular retention. As these microorganisms are consumed by higher trophic levels, boron–toxin complexes could be transferred along the food chain, where changes in pH, ionic strength, or metabolic conditions may modulate complex dissociation or reformation. This dynamic equilibrium provides a plausible chemical mechanism by which tetrodotoxin is accumulated, retained, and redistributed across diverse marine organisms—including invertebrates, fish, and ultimately apex consumers—without requiring de novo toxin biosynthesis at each trophic level.

## 11. Saxitoxins and Their Boron Complexes

Saxitoxin (STX) is an extremely potent neurotoxin produced by marine dinoflagellates as well as freshwater and brackish-water cyanobacteria and is classified as a paralytic shellfish toxin (PST). STX is a highly specific blocker of voltage-gated sodium channels (Na_v_), where it binds to the extracellular pore region and prevents sodium ion influx. By occluding the ion-conducting pathway of these channels, the toxin effectively interrupts the generation and propagation of action potentials in excitable tissues. This inhibition disrupts action potential propagation, leading to profound neurological dysfunction and systemic disturbances affecting the nervous, respiratory, cardiovascular, and gastrointestinal systems. Severe intoxication can result in flaccid paralysis, respiratory failure, and death [251,252,253,254]. Because of its extreme potency and rapid physiological effects, saxitoxin has been widely studied in neurophysiology as a molecular probe for sodium channel function.

Human exposure to STX occurs primarily through the consumption of contaminated shellfish, making it the principal causative agent of paralytic shellfish poisoning (PSP) and a major public health concern worldwide. Shellfish such as mussels, clams, oysters, and scallops can accumulate saxitoxin and its analogs through filter-feeding on toxin-producing microalgae. Consequently, monitoring programs have been established in many coastal regions to detect harmful algal blooms and prevent contaminated seafood from entering the food supply. Beyond its acute toxicity, STX has cascading consequences for food safety, marine ecosystem stability, and coastal economies, particularly in regions impacted by harmful algal blooms (HABs) [255,256,257,258]. Increasing frequency and geographic expansion of HAB events have intensified global concerns regarding STX contamination in marine environments.

**Figure 14:**
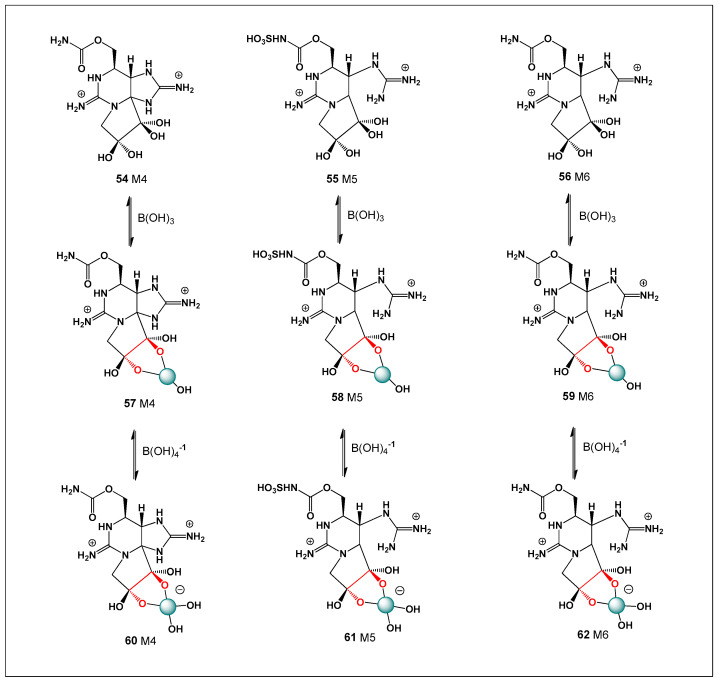
Chemical structures of selected saxitoxin (STX) analogs and a proposed pathway for boron complex formation. Among the more than 50 known saxitoxins, only a limited subset—M4, M5, and M6—contains vicinal 1,2-diol groups capable of coordinating boric acid or borate anions to form reversible boron complexes. Given the relatively high concentration of dissolved boron in marine environments and its pH-dependent speciation, such complexation may enhance toxin stability, solubility, and persistence in seawater. These physicochemical changes could facilitate bioaccumulation in primary consumers (e.g., shellfish) and promote trophic transfer through marine food webs, ultimately increasing exposure risk for higher-level predators, including humans. Like other saxitoxins, these boron-complex-forming derivatives potently block voltage-gated sodium channels, thereby inhibiting sodium ion influx and suppressing nerve impulse transmission, which results in systemic physiological disturbances affecting the nervous, respiratory, cardiovascular, and gastrointestinal systems.

Structurally, STX possesses a compact tricyclic skeleton incorporating a highly functionalized guanidinium-rich framework. This molecular architecture enables strong electrostatic interactions with negatively charged residues within sodium channel binding sites. More than 50 naturally occurring STX analogs have been identified, differing in hydroxylation, sulfation, and carbamate substitution patterns [259,260]. Among STX derivatives, only M4 (**54**), M5 (**55**), and M6 (**56**, see Figure 14) contain vicinal 1,2-diol moieties capable of coordinating boric acid and borate anions. These cis-diol groups provide chemically plausible binding sites for reversible boron complexation, analogous to well-established boron–diol interactions observed in carbohydrates and polyhydroxylated natural products. Formation of such boron complexes (**57**, **58**, **59**, **60**, **61**, and **62**) may enhance aqueous solubility, alter molecular conformation, and potentially facilitate more efficient delivery of these toxins to their biological targets, although the precise physiological implications of this interaction remain to be fully elucidated [261,262,263,264]. Such reversible borate interactions may also influence toxin persistence and transport in marine environments where dissolved boron is abundant.

STX originates from a wide range of biological sources. In marine environments, toxin production is primarily associated with dinoflagellates of the genera *Alexandrium* (at least 10 species), *Pyrodinium bahamense*, *Gymnodinium catenatum*, and *Centrodinium punctatum* [265,266,267]. In freshwater systems, STX is produced by cyanobacteria including *Raphidiopsis brookii*, *Anabaena circinalis*, *Aphanizomenon* spp., *Raphidiopsis raciborskii*, and *Microseira wollei*. To date, at least 15 freshwater cyanobacterial species have been confirmed to possess the genetic machinery required for STX biosynthesis [268,269,270,271]. The widespread occurrence of these organisms demonstrates that saxitoxin production is not restricted to a single ecological niche. Instead, toxin biosynthesis appears to be distributed among diverse microbial taxa capable of thriving in both marine and freshwater habitats. This ecological diversity contributes to the global distribution of saxitoxin and complicates efforts to predict or control harmful algal bloom events.

Symptoms typically appear within a few minutes to several hours after ingestion and include nausea, vomiting, oral and facial paresthesia, visual disturbances, myalgia, progressive muscle weakness, and paralysis. In severe cases, respiratory muscle failure leads to asphyxiation and death. Globally, approximately 2000 cases of paralytic shellfish poisoning (PSP) are reported annually, with mortality rates reaching up to 15% in severe outbreaks, underscoring the urgent need for continued investigation into STX chemistry, bioavailability, and environmental behavior [272,273,274,275].

In the context of marine boron availability and pH-dependent boron speciation, the capacity of selected STX analogs to form reversible boron complexes introduces an additional dimension to understanding toxin transport, bioaccumulation, and effective toxicity in marine food webs. This aspect aligns STX with other vicinal diol–containing marine toxins and antibiotics discussed in this review and highlights boron coordination chemistry as a potentially underappreciated factor influencing the ecological and biological impact of marine natural products [276,277].

## 12. Azaspiracid Toxins and Their Boron Complexes

**Figure 15:**
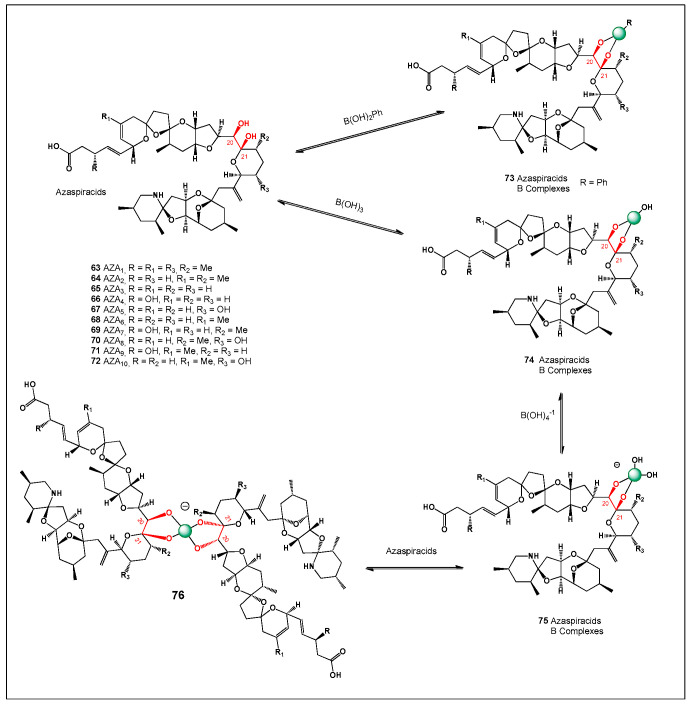
Chemical structures of azaspiracids and the proposed pathway for boron complex formation. Marine dinoflagellates of the genus Azadinium are the primary biological source of azaspiracids (AZAs), a group of polyether marine toxins. AZAs accumulate in suspension-feeding shellfish and other seafood, causing azaspiracid poisoning (AZP) in humans, which is characterized by gastrointestinal symptoms such as diarrhea, nausea, vomiting, and abdominal pain, and may include neurological disturbances. Prolonged or repeated exposure has been associated with tissue damage in organs such as the liver and lungs. The presence of vicinal diol motifs in selected AZA structures suggests a plausible pathway for reversible boron complexation under marine conditions, potentially influencing toxin stability, bioavailability, and trophic transfer.

Compared with the extensive knowledge available on azaspiracid (AZA, Figure 15) structures, detection methods, and toxicology, clarification of AZA etiology lagged behind for many years. Early observations of seasonal and episodic AZA accumulation in suspension-feeding bivalves suggested a planktonic origin, and the polyether structure of AZAs (63–71) initially pointed to dinoflagellates as likely producers [278,279,280].

These compounds belong to a structurally complex class of polyether marine toxins characterized by multiple cyclic ether rings and extensive oxygenation. Accordingly, Yasumoto [281] first proposed a species of the heterotrophic dinoflagellate genus *Protoperidinium* as the AZA source based on LC–MS analysis of net-haul phytoplankton, later identified as *Protoperidinium crassipes*. However, this association remained controversial, as AZA production could not be confirmed in field surveys or laboratory cultures, and *P. crassipes* is known to be heterotrophic, feeding on other dinoflagellates, raising the possibility of trophic accumulation rather than de novo toxin synthesis [282,283]. This uncertainty highlighted the complexity of tracing toxin biosynthesis in marine food webs. It also emphasized the importance of combining ecological observations with analytical chemistry to identify the true biological sources of marine toxins.

This uncertainty was clarified during a research cruise in the North Sea, where AZAs were detected in plankton samples lacking *P. crassipes*, in predatory ciliates (*Favella ehrenbergii*), and predominantly in small-sized (<20 μm) plankton fractions. These findings suggested that the primary toxin producers were small planktonic organisms that had previously escaped detection. Subsequent investigations led to the isolation of a small dinoflagellate capable of producing AZA1 and AZA2 in axenic culture, later described as *Azadinium spinosum*, a new species within a newly established genus. Since its discovery, the genus *Azadinium* has rapidly expanded to include six species, along with the closely related genus *Amphidoma* [284]. While *A. spinosum* strains consistently produce AZA1, AZA2, and AZA7, other species initially appeared non-toxigenic until the discovery of additional AZA analogs revealed substantial chemical diversity across species and strains. Notably, *A. poporum* produces a wide range of AZAs with pronounced strain-dependent variability, while some species remain negative for known AZAs, possibly due to the presence of yet-undetected analogs [285]. This expanding diversity of AZA-producing organisms highlights the dynamic and evolving nature of toxin biosynthesis in marine ecosystems.

Phenylboronic acid, when reacting with azaspiracid toxins (**63**–**72**), forms a strong boron complex (**73**), as shown by Beach and co-workers [250]. Similarly, azaspiracid toxins (**63**–**72**) react with boric acid or boron anion to form complexes (**74**–**76**).

Overall, the growing diversity of AZA-producing species underscores the importance of understanding both shared and distinguishing biological traits to support accurate species identification, monitoring programs, and early-warning systems for harmful algal blooms [286,287].

*Azadinium* is a genus of small marine dinoflagellates (microalgae) belonging to the family Amphidomataceae and is best known as the primary biological source of azaspiracid (AZA) marine biotoxins. Species of *Azadinium* are widely distributed throughout the world’s oceans, including the Atlantic, Mediterranean, and North Seas. These microorganisms are typically very small (<20 μm) and can therefore be easily overlooked in routine plankton surveys. The genus currently comprises more than 15 described species, of which *Azadinium spinosum* (the type species), *A. poporum*, *A. dexteroporum*, and *A. obesum* are the most extensively studied. Several species—most notably *A. spinosum*—produce azaspiracids, which bioaccumulate in suspension-feeding bivalve mollusks such as mussels and oysters, posing a significant risk to seafood safety and human health [278,279,280,281,282,287]. Because of their small size and cryptic morphology, accurate identification of *Azadinium* species often requires molecular genetic analysis in addition to conventional microscopy.

Although *Azadinium* was initially described from the North Sea, accumulating evidence indicates that the genus is widely distributed on a global scale. In the North Sea region alone, five described species have been reported. Records from the Scottish and Irish Atlantic coasts, northern Norway, and the Shetland Islands suggest that *Azadinium* extends into more northern North Atlantic and Arctic waters. This is further supported by light-microscopy observations of *Azadinium* spp. in plankton samples from the Irminger Sea between Greenland and Iceland, although species-level identification remains unresolved. Along the French Atlantic coast, several *Azadinium* species—including *A. caudatum* and *A. poporum*—have been detected, with additional taxa currently under taxonomic evaluation [282,283,284,285]. These findings indicate that *Azadinium* populations may be more widely distributed than previously assumed. Ongoing environmental DNA surveys are expected to further expand our understanding of the geographic distribution of this genus.

The type species *A. spinosum* has been isolated from multiple European Atlantic locations, including Scotland, Denmark, Ireland, and the Shetland Islands. SEM observations also suggest its presence in Pacific waters off Mexico, implying a broader distribution, although molecular confirmation is lacking. *A. obesum* has so far been reported only from the Scottish coast, while *A. caudatum*—easily recognizable by light microscopy—has the best-documented biogeography, spanning the North Sea, Atlantic coasts of Europe, the Mediterranean, and the French Atlantic. *A. polongum* has been reported exclusively from the Shetland Islands and appears adapted to colder northern waters [282,283,284,288,289,290]. The diversity of ecological niches occupied by these species reflects their ability to adapt to different environmental conditions. Such adaptability likely contributes to the persistence and spread of azaspiracid-producing populations in marine ecosystems.

*Azadinium poporum* is the most geographically widespread species, with numerous strains isolated from the western Pacific, including Korean and Chinese coastal waters, where substantial genetic diversity has been documented [291]. Genetic analyses have revealed considerable intra-species variation, suggesting the presence of multiple ecotypes adapted to distinct environmental conditions. The AZA-producing species *Amphidoma languida*, initially isolated from Ireland, is likely more widely distributed, with morphological and molecular evidence indicating its presence in the eastern Atlantic and North Sea [284,292]. In addition, *Azadinium* spp. have been reported from the southern Atlantic off Argentina and from Mediterranean and Black Sea regions, further underscoring the global distribution and ecological relevance of this toxin-producing dinoflagellate group [285,292]. The expanding recognition of *Azadinium* species in diverse marine environments highlights their importance as contributors to harmful algal blooms and toxin contamination events. Continued monitoring of these organisms is therefore essential for improving predictive models of azaspiracid occurrence and mitigating risks to seafood safety and marine ecosystem health.

## 13. Pectenotoxins and Their Boron Complexes

**Figure 16:**
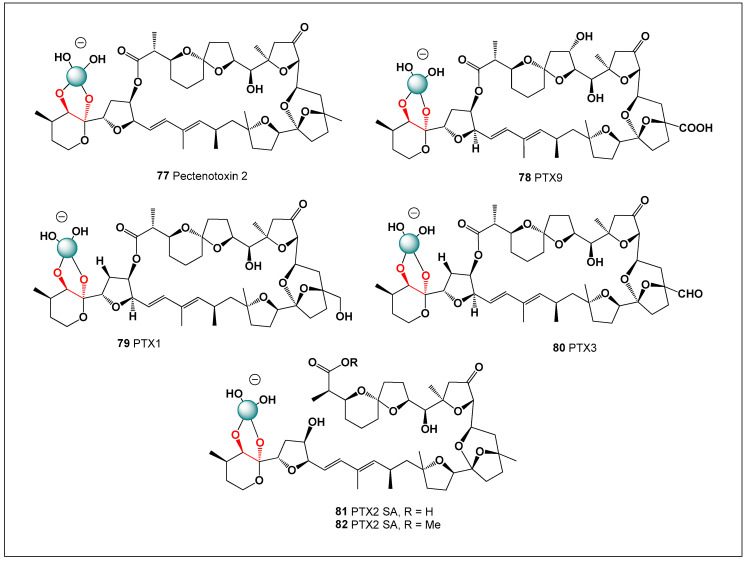
Chemical structures of selected pectenotoxins produced by marine dinoflagellates and accumulated in scallops. These toxins contain vicinal hydroxyl groups at the C-2 and C-3 positions (red marked), which can serve as coordination sites for boric acid or borate anions under marine conditions. Such reversible boron complexation may enhance toxin solubility and persistence in aquatic environments. The overall toxicity is primarily associated with the pectenotoxin framework itself; boron species may modulate bioavailability or transport rather than acting as independent toxic agents. Compared with AZAs and TTX, PTX boron complexation is likely mediated by distributed polyether oxygen arrays rather than discrete cyclic borate esters, suggesting a weaker but persistent mode of coordination.

Marine polyether metabolites known as pectenotoxins (PTXs) are a group of shellfish-associated toxins originally linked to diarrhetic shellfish poisoning (DSP), particularly in scallops such as *Patinopecten yessoensis*. PTXs are produced primarily by planktonic dinoflagellates of the genus *Dinophysis* (including *D. acuta*, *D. fortii*, *D. acuminata*, *D. norvegica*, *D. mitra*, and *D. caudata*) and have also been detected in benthic species such as *Prorocentrum lima*. Structurally, pectenotoxins belong to a class of large polyether macrolides characterized by multiple fused cyclic ether rings and extensive oxygenation. Experimental studies demonstrate that PTXs exhibit pronounced cytotoxicity, induce hepatocyte damage, and promote tumor development in cellular and animal models [293,294]. These toxins are known to disrupt cytoskeletal organization, particularly by interfering with actin filament dynamics in eukaryotic cells. However, despite extensive investigation of their acute toxicity, the chronic toxicological effects of PTXs and their long-term implications for human health remain poorly understood. Figure 16 illustrates the proposed boron complexes (77–82, Figure 16) of PTXs, highlighting the potential role of vicinal diol motifs in boron coordination, which may influence toxin stability, bioavailability, and accumulation in marine food webs. The presence of multiple hydroxyl groups within PTX structures provides potential coordination sites for boron species present in seawater.

In contrast to azaspiracids (AZAs) and tetrodotoxin (TTX), for which boron complexation is supported by the presence of well-defined vicinal 1,2-diol motifs and, in the case of TTX, by experimental isolation of boron-associated forms, pectenotoxins (PTXs) represent an intermediate case. Like AZAs, PTXs are large polyether metabolites containing multiple oxygen-rich domains capable of coordinating boron species under marine conditions. However, unlike TTX or selected saxitoxin analogs (e.g., M4–M6), PTXs lack a single dominant diol site and instead present distributed polyol environments that may enable multidentate, reversible borate coordination. Such interactions may occur transiently in aqueous environments where boric acid and borate anions are present at significant concentrations. This mode of interaction is likely weaker and more dynamic than the cyclic borate esters proposed for TTX and specific STXs but may still contribute to enhanced stability, altered aggregation behavior, and prolonged persistence in marine food webs. Furthermore, reversible boron coordination could influence the conformational flexibility of PTX molecules and their interactions with biological membranes or intracellular targets. These considerations suggest that boron–polyether interactions may represent an additional factor influencing the environmental fate and biological activity of marine toxins.

Collectively, these comparisons suggest that boron complexation of marine toxins exists along a continuum of structural specificity, ranging from highly defined diol–borate interactions (TTX, STX M-series) to more diffuse polyether coordination (AZAs, PTXs), with potential consequences for bioaccumulation, trophic transfer, and effective toxicity.

## 14. CTXs and Gambierones and Their Boron Complexes

A group of lipid-soluble polyether compounds known as ciguatoxins (CTXs) are highly potent ichthyotoxins produced by the benthic dinoflagellate *Gambierdiscus toxicus* [295,296,297]. These toxins are among the most complex marine natural products, characterized by large ladder-like polyether frameworks composed of multiple fused ether rings. The principal toxin, ciguatoxin, was first isolated from the liver of a moray eel collected off the Hawaiian Islands. *G. toxicus*, an epiphytic dinoflagellate associated with coral reef ecosystems, represents the primary source of ciguatoxins, which bioaccumulate and biomagnify through marine food webs, ultimately reaching high concentrations in predatory fish [298,299]. Herbivorous reef fish typically acquire these toxins by grazing on algae and detritus colonized by toxin-producing dinoflagellates. Consumption of contaminated fish from tropical and subtropical regions leads to ciguatera fish poisoning (CFP), one of the most common forms of non-bacterial seafood poisoning worldwide [300,301]. Because CTXs are highly stable and resistant to heat, freezing, and conventional cooking methods, contaminated seafood remains hazardous even after food preparation.

Species of the benthic dinoflagellate genera *Gambierdiscus* and *Fukuyoa* produce a wide array of ladder-shaped polyether metabolites, including gambierol, gambieric acids, maitotoxins, ciguatoxins (CTXs), and gambierones [302,303]. These structurally related metabolites display a broad range of biological activities affecting ion channels and membrane physiology. Among these, gambierone and 44-methylgambierone belong to a distinct class of sulfated polyether compounds that have been identified across multiple species of *Gambierdiscus* and *Fukuyoa*, as well as in the related benthic dinoflagellate *Coolia tropicalis* [304,305]. The chemical diversity of these compounds reflects the remarkable biosynthetic capabilities of benthic dinoflagellates inhabiting coral reef ecosystems. Many of these metabolites possess multiple hydroxyl groups that contribute to their chemical reactivity and interactions with biological membranes.

**Figure 17:**
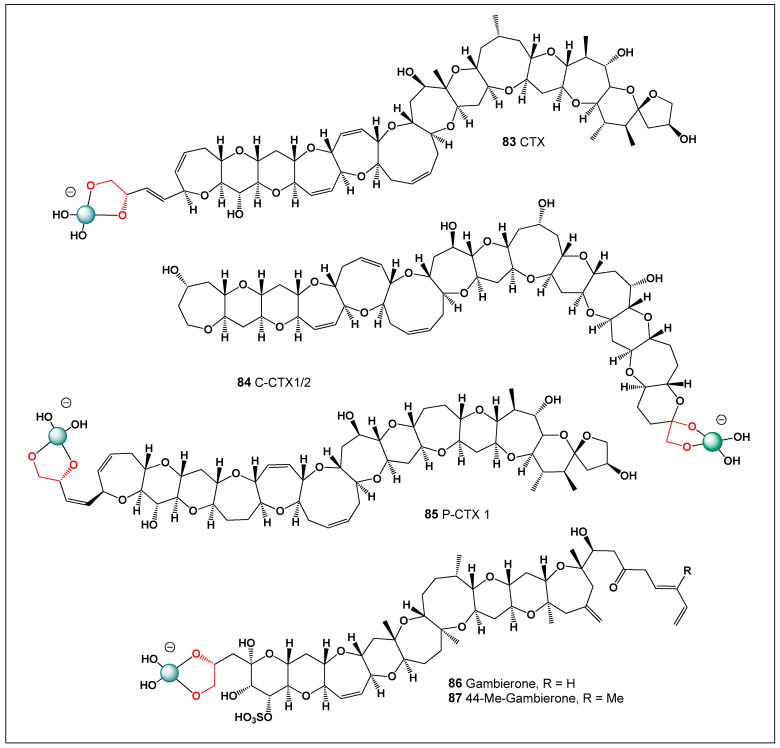
CTXs and Gambierones produced by dinoflagellates, and some other microorganisms, contain terminal 1,2-diol groups that are capable of forming boron complexes.

Ciguatoxins (83–85; structures shown in Figure 17) are highly potent polyether neurotoxins and the primary causative agents of ciguatera fish poisoning (CFP), currently the most widespread marine biotoxin–related foodborne illness worldwide. CFP arises from the consumption of tropical and subtropical fish and marine invertebrates contaminated with CTXs that bioaccumulate and biomagnify through coral reef food webs. At extremely low concentrations, CTXs act as strong activators of voltage-gated sodium channels, leading to persistent depolarization of excitable membranes and severe neurological, cardiovascular, and gastrointestinal symptoms [297,298,299,300]. These physiological effects result in a wide spectrum of clinical manifestations, including paresthesia, temperature reversal sensations, gastrointestinal distress, and cardiovascular disturbances. Because CTXs remain tightly associated with lipid-rich tissues, toxin accumulation is particularly pronounced in predatory reef fish occupying higher trophic levels.

Structurally, ciguatoxins and gambierones possess multiple hydroxylated polyether segments, including vicinal and pseudo-vicinal diol motifs embedded within their ladder-like frameworks. These oxygen-rich domains provide potential coordination sites for metal ions and metalloid species in marine environments. By analogy with azaspiracids (AZAs), pectenotoxins (PTXs), saxitoxin derivatives, and tetrodotoxin (TTX), these diol-rich regions represent plausible coordination sites for boric acid or borate anions (83–87, structures shown in Figure 17) under marine conditions [306]. Given the relatively high concentration of dissolved boron in seawater and the pH-dependent equilibrium between boric acid and borate species, reversible formation of boron–polyether complexes is chemically feasible. Such interactions may influence the conformational stability, aggregation behavior, and environmental persistence of these toxins. In addition, boron coordination could potentially modulate the interaction of these polyether toxins with biological membranes or ion channel targets. These considerations further support the hypothesis that boron–toxin interactions may play an underappreciated role in the environmental chemistry and biological activity of marine polyether toxins.

Such complexation is expected to be non-covalent and dynamic, potentially influencing toxin solubility, aggregation behavior, membrane partitioning, and transport through aqueous and biological interfaces. In the context of trophic transfer, boron coordination may facilitate the persistence and mobility of CTXs and gambierones within planktonic prey, reef fish, and higher predators, thereby contributing to their efficient bioaccumulation and biomagnification. While direct experimental evidence for stable boron–CTX or boron–gambierone complexes remains limited, the structural and environmental parallels with other marine toxins discussed in this review strongly support this hypothesis [306,307].

Gambierones exhibit CTX-like interactions with sodium channels but with substantially lower potency and minimal acute toxicity in mouse intraperitoneal bioassays [308,309,310]. Nonetheless, potential boron complexation may partially compensate for lower intrinsic activity by enhancing aqueous stability or delivery efficiency. Consequently, gambierones—together with their boron-complexed forms—may serve not only as contributors to overall toxin profiles but also as chemical biomarkers for monitoring Gambierdiscus dominance and associated toxicological risk in coral reef ecosystems [311,312,313,314,315].

## 15. Natural Toxins Featuring 1,3-Diol Structural Motifs

Several natural toxins contain 1,3-diol moieties embedded within aromatic or polyhydroxylated frameworks, which can participate in reversible coordination with boron species or other Lewis acids. One of the simplest structural motifs illustrating this arrangement is 1,8-naphthalenediol (naphthalene-1,8-diol)**,** an aromatic compound in which two hydroxyl groups are positioned in a 1,3-relationship across the fused ring system [2,25,31]. Such structures occur as building blocks or derivatives in several classes of fungal and microbial secondary metabolites, including naphthoquinone toxins, melanin precursors, and related phenolic metabolites produced by phytopathogenic fungi. In these systems, the 1,3-diol configuration can participate in intramolecular hydrogen bonding and metal or metalloid coordination, which may influence molecular stability and reactivity. More complex toxins containing analogous aromatic dihydroxy arrangements are found among fungal polyketides such as viriditoxin derivatives, borolithochrome-related metabolites, and other naphthalene-based phytotoxins, where hydroxylated aromatic rings contribute to redox activity and phototoxic properties. The presence of 1,3-dihydroxy aromatic systems can facilitate interactions with metal ions and potentially with boric acid or borate species through the formation of cyclic or chelated complexes. Although such interactions are generally weaker than those formed by *cis*-1,2-diols, the rigid aromatic framework of compounds like 1,8-naphthalenediol may stabilize six-membered borate coordination motifs under suitable conditions [25,31,49,50]. These structural features highlight the broader relevance of 1,3-diol motifs in toxin chemistry, where they may contribute not only to biological activity but also to environmental behavior, molecular aggregation, and interactions with metalloid species present in natural ecosystems. Detailed examples of toxins incorporating aromatic 1,3-diol units and their potential chemical implications are discussed in the following sections.

### 15.1. Viriditoxin and Its Boron Complexes

Viriditoxin (**88**) is a well-known fungal secondary metabolite and potent mycotoxin produced by several species of the genera *Aspergillus* (e.g., *A. viridinutans*, *A. brevipes*) and *Paecilomyces variotii*. First isolated in 1971, viriditoxin belongs to the class of binaftopyranone polyketides and exhibits a distinctive axially chiral (atropisomeric) helical structure, which contributes to its biological activity and stereochemical stability [316,317,318,319]. The compound displays strong antibacterial activity, particularly against Gram-positive bacteria, including antibiotic-resistant strains such as methicillin-resistant *Staphylococcus aureus* (MRSA). Mechanistically, viriditoxin targets the bacterial cytoskeletal protein FtsZ, a tubulin homolog essential for bacterial cytokinesis [320,321]. By inhibiting the GTPase activity of FtsZ, viriditoxin prevents formation of the Z-ring required for cell division, ultimately suppressing bacterial proliferation. Beyond its antibacterial effects, viriditoxin also exhibits notable antitumor properties, inducing apoptosis in leukemia and lymphoma cells through disruption of mitochondrial oxidative phosphorylation. In ovarian cancer cells (SK-OV-3 line), the toxin has been shown to stabilize microtubule polymers in a manner analogous to paclitaxel, resulting in arrest of the cell cycle at the G_2_/M phase. Despite these promising biological activities, viriditoxin is also toxic to mammals, with a reported LD_50_ of approximately 2.8 mg/kg in mice following intraperitoneal administration [322,323,324].

Structurally, viriditoxin contains multiple phenolic hydroxyl groups arranged in aromatic diol environments, including motifs analogous to 1,3-dihydroxy (resorcinol-type) systems within its naphthalene-derived framework. These hydroxylated aromatic domains provide potential coordination sites for boric acid or borate anions, enabling the formation of reversible boron–viriditoxin complexes through borate ester interactions. Although such complexes have not yet been extensively characterized experimentally, the geometry of the phenolic diol units is chemically compatible with the formation of cyclic or multidentate borate coordination structures.

In aqueous environments containing dissolved boron, such interactions could influence the conformation, aggregation behavior, and redox properties of the viriditoxin molecule. Because viriditoxin already possesses a rigid atropisomeric structure, boron coordination may further stabilize specific conformations or alter its interaction with biological targets such as FtsZ or microtubules. These considerations suggest that boron complexation could represent an additional factor influencing the environmental stability and biological activity of viriditoxin and related fungal polyphenolic toxins. Consequently, viriditoxin provides an intriguing example of a 1,3-diol–containing natural toxin capable of potential boron coordination, illustrating how metalloid interactions may modulate the chemistry and toxicology of fungal secondary metabolites.

**Figure 18:**
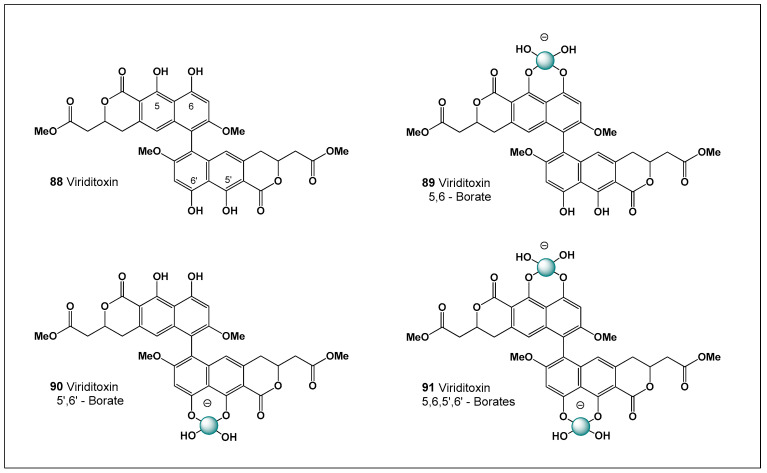
Chemical structures of viriditoxin and its three principal types of boron-containing complexes: mono-spiroborate (**89**), di-spiroborate (**90**), and tri-spiroborate (**91**) isomers. These complexes arise from coordination of borate with phenolic hydroxyl groups within the polyaromatic viriditoxin framework, forming tetrahedral spiroborate centers. The number of coordinated boron atoms reflects the availability of dihydroxy binding sites and illustrates the potential for progressive boron complexation in polyphenolic fungal metabolites.

Because boron commonly forms tetrahedral spiroborate complexes with polyphenolic ligands, viriditoxin can theoretically generate several distinct boron-associated structures depending on the number of coordinating hydroxyl groups involved. On this basis, viriditoxin may form three principal types of boron-containing complexes: mono-spiroborate (**89**), di-spiroborate (**90**), and tri-spiroborate (**91**) isomers (Figure 18).

In the mono-spiroborate form, a single boron atom coordinates with two oxygen atoms derived from one dihydroxy aromatic fragment of the viriditoxin molecule. This interaction leads to the formation of a spirocyclic borate ester, where the boron atom adopts a tetrahedral geometry and bridges the two phenolic oxygen atoms through B–O bonds. Such complexes are commonly observed in polyphenolic systems and may stabilize specific conformations of the molecule by restricting rotation of aromatic rings. In viriditoxin, formation of a mono-spiroborate complex would likely occur at the most sterically accessible phenolic diol site within the naphthopyranone framework. This interaction could influence the electronic distribution of the aromatic system and potentially modify the molecule’s biological reactivity or binding interactions with proteins such as the bacterial cytoskeletal protein FtsZ.

More extensive coordination may lead to formation of di-spiroborate complexes, in which two independent boron atoms simultaneously interact with separate phenolic diol domains within the viriditoxin structure. In this configuration, each boron center forms its own cyclic borate ester, resulting in a molecule that contains two boron–oxygen coordination units. Because viriditoxin consists of two coupled naphthopyranone subunits arranged in an atropisomeric configuration, each half of the molecule could potentially participate in boron binding. Such di-spiroborate structures would further rigidify the molecular framework and might alter intermolecular aggregation or stacking interactions commonly observed among polyaromatic fungal metabolites. In addition, coordination of two boron centers could modify the polarity and solubility of viriditoxin, particularly in aqueous environments containing dissolved boric acid.

The most extensive boron coordination scenario involves the formation of tri-spiroborate complexes, where three boron atoms are incorporated through interactions with multiple hydroxylated regions of the molecule. In this arrangement, several phenolic oxygen atoms participate simultaneously in borate ester formation, creating a network of boron–oxygen linkages distributed across the viriditoxin scaffold. Such structures would represent highly coordinated boron complexes analogous to those observed in certain polyphenolic natural products and synthetic borate receptors. The resulting complexes would likely exhibit significant changes in conformational rigidity, electronic distribution, and supramolecular behavior. Formation of tri-spiroborate species may be favored in environments with relatively high concentrations of borate ions, such as marine or alkaline conditions.

The existence of mono-, di-, and tri-spiroborate viriditoxin complexes therefore represents a plausible chemical scenario arising from the molecule’s polyphenolic architecture. These potential complexes illustrate how boron coordination can introduce additional structural diversity to naturally occurring toxins and secondary metabolites. If such complexes occur in biological or environmental systems, they could influence the stability, transport, aggregation behavior, and biological activity of viriditoxin. Moreover, boron coordination might modulate the interaction of the toxin with its known molecular targets, including bacterial FtsZ proteins or eukaryotic cytoskeletal components. Consequently, viriditoxin provides an intriguing example of how polyphenolic fungal toxins containing diol-like motifs may engage in boron coordination chemistry, expanding the conceptual framework of metalloid interactions in natural product toxicology.

### 15.2. Natural Borolithochrome Complexes

Red algae represent one of the most elegant and visually striking groups of marine organisms, distinguished by their diverse pigmentation and morphological forms. These algae are among the oldest known photosynthetic eukaryotes, with fossil evidence indicating that they existed as early as the Cretaceous period [325,326,327]. Red algae share several important biological features with cyanobacteria (blue-green algae), including similarities in photosynthetic pigments, thylakoid organization, and the composition of cellular reserve materials [328,329,330]. In addition to their ecological importance, red algae are rich sources of chemically diverse metabolites, including unusual fatty acids [331,332,333], polar lipids, structurally complex polysaccharides, and halogenated compounds with antifouling and antimicrobial activities [333,334,335,336]. Numerous other bioactive secondary metabolites have also been identified from red algae, reflecting the remarkable metabolic diversity of this group. These natural products contribute to chemical defense, ecological competition, and environmental adaptation in marine ecosystems. As a result, red algae have become important targets in natural product chemistry and marine biotechnology research.

*Solenopora jurassica* is a fossil calcareous organism traditionally interpreted as a red alga that was widely distributed in shallow marine environments during the Jurassic period [337,338]. The organism formed distinctive nodular or massive carbonate structures composed of concentrically layered calcified tissues, which are commonly preserved in Jurassic limestone deposits throughout Europe and other regions. Because of its abundant fossil record and characteristic morphology, *S. jurassica* has long been considered an important component of ancient reef and lagoonal ecosystems. Early paleobotanical studies classified *Solenopora* within the Rhodophyta (red algae) due to similarities in calcification patterns and overall morphology. However, later investigations have suggested that some *Solenopora*-like fossils may instead represent calcifying microbial consortia or bacteria–alga associations rather than true algal organisms. This uncertainty has stimulated considerable interest in the biochemical composition of these fossils, particularly the presence of unusual organic pigments such as borolithochromes preserved within the calcareous matrix. The discovery of these boron-containing pigments in *S. jurassica* has provided valuable insight into the chemical ecology of ancient marine systems and suggests that boron-associated secondary metabolites may have existed in marine microorganisms for hundreds of millions of years. Consequently, *Solenopora jurassica* represents not only an important paleontological species but also a key model for studying the preservation and evolution of complex natural products in the fossil record [339,340,341].

**Figure 19:**
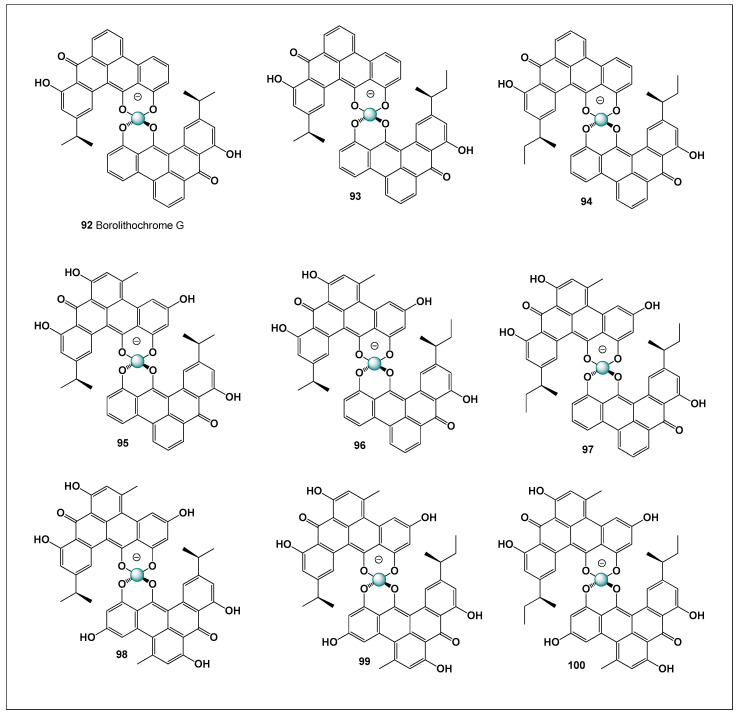
Natural boron-containing organic pigments from a Jurassic red alga. These are polyketide-derived spiroborate pigments. The structures illustrate the coordination of a central boron atom by phenolic oxygen atoms from two polyaromatic ligands, forming stable spiroborate complexes. Such pigments represent rare examples of naturally occurring boron-containing aromatic polyketides preserved in the fossil record. Their discovery provides important insight into the long-term stability and evolutionary history of boron-associated natural products in ancient marine environments.

A group of researchers from the University of Göttingen and the University of Linz determined the structure and origin of Jurassic-period borolithochromes. Borolithochromes (**92**–**100**, Figure 19) are organic biomolecules derived from the Jurassic fossil organism traditionally assigned to the putative red alga *Solenopora jurassica* [339]. These pigments were identified as spiroborate complexes containing two pentacyclic sec-butyl-trihydroxy-methyl-benzo[gh]tetraphenone ligands and related less-substituted derivatives.

Structurally, borolithochromes are unique spiroborates in which a central boron atom coordinates two phenolic ligands, forming highly stable boric acid ester structures. This class of compounds represents the first reported example of naturally occurring boronated aromatic polyketides preserved in the fossil record. Their remarkable structural similarity to modern secondary metabolites such as clostrubin A suggests that borolithochromes may originate from ancient microbial producers rather than from algae themselves. Consequently, these pigments may represent fossilized bacterial secondary metabolites preserved within calcareous algal deposits. The borolithochromes are therefore characterized as complex spiroborates with two phenolic moieties acting as boron ligands, forming a unique class of fossil organic pigments [339,340,341]. These compounds provide rare insight into the evolution of boron-containing natural products and highlight the long-standing role of boron coordination chemistry in biological systems.

## 16. Conclusions

This review highlights boron as a chemically subtle yet biologically powerful element whose coordination chemistry underlies a true pharmacological duality. Across antibiotics and toxins, boron does not act merely as a passive trace element; rather, it functions as an active molecular modulator, capable of amplifying biological effects through reversible complex formation with oxygen-rich organic frameworks. The central unifying theme emerging from this work is that boron’s impact is context-dependent, governed by molecular structure, stereo-chemical accessibility, pH-dependent speciation, and environmental availability.

In the case of antibiotics, boron complexation frequently confers clear benefits. Boron-containing polyketide antibiotics such as boromycin, aplasmomycin, tartrolons, and hyaboron incorporate boron directly into their molecular architecture, where it contributes to ionophoric behavior, membrane disruption, and potent antibacterial or antiparasitic activity. Beyond intrinsically borated molecules, many clinically important antibiotics—including tetracyclines, polyene macrolides (amphotericin B, fungichromin, nystatin), rifamycins, and sorangicin A—possess vicinal 1,2-diol motifs that enable reversible borate complexation. Such interactions can modulate aggregation, improve aqueous solubility, alter membrane affinity, and fine-tune selectivity toward Gram-positive bacteria or fungal membranes. In these contexts, boron acts as a beneficial enhancer, improving pharmacokinetic properties and, in some cases, reducing toxicity through controlled aggregation or formulation effects.

The presence of 1,3-diol motifs in natural toxins and secondary metabolites represents an additional structural feature capable of participating in boron coordination chemistry. Although 1,3-diols generally form weaker complexes with boric acid than cis-1,2-diols, they can still generate six-membered borate ester structures or participate in multidentate coordination within polyhydroxylated frameworks. Such interactions may influence molecular conformation, aggregation behavior, and the environmental stability of these compounds, particularly in boron-rich marine systems. Consequently, 1,3-diol–containing natural products represent an important but still underexplored class of molecules in which boron coordination may modulate biological activity and ecological persistence.

Conversely, in the realm of toxins, the same chemical principles lead to markedly different biological outcomes. Marine and freshwater toxins such as tetrodotoxin, selected saxitoxin derivatives, azaspiracids, pectenotoxins, ciguatoxins, and gambierones often contain diol-rich or polyether frameworks capable of forming boron complexes under physiological or marine conditions. Given the relatively high concentration of boron in seawater and its pH-dependent speciation, such complexation is chemically plausible and likely biologically relevant. Boron coordination in these systems may enhance toxin solubility, stabilize bioactive conformations, facilitate transport across membranes, and promote bioaccumulation and trophic transfer along marine food chains. Here, boron acts as a potentiator of toxicity, amplifying the harmful effects of already potent neurotoxins and cytotoxins.

Taken together, these observations demonstrate that boron represents a double-edged element in chemical biology. The same coordination chemistry that enhances therapeutic efficacy in antibiotics can equally intensify toxicity in natural poisons. Whether boron’s influence is beneficial or detrimental depends critically on molecular architecture, stereochemical volume, coordination geometry, and environmental conditions such as pH and ion composition. This duality underscores the necessity of considering boron not only as a micronutrient or geochemical constant, but as an active participant in bioorganic chemistry and pharmacology.

Ultimately, this review provides a unified conceptual framework linking boron availability, molecular structure, and biological outcome. Recognizing boron’s central role in both antibiotic enhancement and toxin amplification opens new avenues for rational drug design, improved formulations, and a deeper understanding of toxin dynamics in marine and terrestrial ecosystems. Boron’s chemistry, when properly harnessed, can serve human health—yet when embedded in toxic frameworks, it becomes a powerful driver of biological harm.

## Data Availability

No new data were created or analyzed in this study. Data sharing is not applicable to this article.
